# A single site ruthenium catalyst for robust soot oxidation without platinum or palladium

**DOI:** 10.1038/s41467-023-42935-7

**Published:** 2023-11-06

**Authors:** Yuanfeng Li, Tian Qin, Yuechang Wei, Jing Xiong, Peng Zhang, Kezhen Lai, Hongjie Chi, Xi Liu, Liwei Chen, Xiaolin Yu, Zhen Zhao, Lina Li, Jian Liu

**Affiliations:** 1 State Key Laboratory of Heavy Oil Processing, Key Laboratory of Optical Detection Technology for Oil and Gas, China University of Petroleum, Beijing, 102249 P. R. China; 2 School of Chemistry and Chemical, In-situ Center for Physical Sciences, Shanghai Jiao Tong University, 200240 Shanghai, P. R. China; 3 State Key Laboratory for Structural Chemistry of Unstable and Stable Species, Beijing National Laboratory for Molecular Sciences (BNLMS), Institute of Chemistry, Chinese Academy of Sciences, Beijing, 100190 China; 4 Shanghai Synchrotron Radiation Facility, Shanghai Advanced Research Institute, Shanghai, China

**Keywords:** Heterogeneous catalysis, Pollution remediation, Materials for energy and catalysis

## Abstract

The quest for efficient non-Pt/Pd catalysts has proved to be a formidable challenge for auto-exhaust purification. Herein, we present an approach to construct a robust catalyst by embedding single-atom Ru sites onto the surface of CeO_2_ through a gas bubbling-assisted membrane deposition method. The formed single-atom Ru sites, which occupy surface lattice sites of CeO_2_, can improve activation efficiency for NO and O_2_. Remarkably, the Ru_1_/CeO_2_ catalyst exhibits exceptional catalytic performance and stability during auto-exhaust carbon particle oxidation (soot), rivaling commercial Pt-based catalysts. The turnover frequency (0.218 h^−1^) is a nine-fold increase relative to the Ru nanoparticle catalyst. We further show that the strong interfacial charge transfer within the atomically dispersed Ru active site greatly enhances the rate-determining step of NO oxidation, resulting in a substantial reduction of the apparent activation energy during soot oxidation. The single-atom Ru catalyst represents a step toward reducing dependence on Pt/Pd-based catalysts.

## Abstract (web summary)

Here, the authors report a study exploring surface lattice-confined single-atom ruthenium sites which demonstrate remarkable stability and exceptional catalytic performance for auto-exhaust purification, making them a promising alternative to costly Pt/Pd-based catalysts.

## Introduction

Auto-exhaust carbon particles (mainly containing soot) constitute a major source of atmospheric pollution, leading to severe environmental and health problems^1,2^. To address this problem effectively, a catalytic after-treatment technique combining particulate filters and oxidation catalyst has been adopted as the most effective strategy^3,4^. The success of this approach heavily relies on high-efficiency catalysts that facilitate soot oxidation at lower temperature range^5^. Researchers have explored numerous high-efficient catalysts for soot oxidation, including precious metal and metal oxides^6–8^. Notably, platinum (Pt) and palladium (Pd) metals remain essentially active components in commercial catalysts for soot purification, with their usage exceeding 42 % of the global demand amount, as reported in the Pgm market report (May 2023) by Johnson Matthey. This high reliance on Pt/Pd significantly contributes to the costly nature of auto-exhaust after-treatment systems. Developing high-efficiency, non-Pt/Pd catalysts with lower costs for soot oxidation presents a challenging task^9^. While the cost of ruthenium (Ru) metal is merely a third of Pt/Pd, the creation of Ru-based catalysts exhibiting both high activity and stability has been rarely reported in vehicle catalysts^10,11^. The primary issue lies in the volatility of Ru oxides at higher temperatures^12,13^. Hence, designing and preparing a robust and cost-effective Ru-based catalyst for auto-exhaust applications, capable of inhibiting volatile Ru at elevated temperatures, holds great significance in replacing Pt/Pd-based catalysts in the field of soot purification. Studies have found that the strong interactions between Ru and CeO_2_ in the Ru/CeO_2_ catalyst can enhance the catalytic activity and the thermal stability during soot oxidation reaction^14^. Therefore, employing a strong metal-support interaction (SMSI) emerges as a reasonable strategy to enhance the stability of Ru-based catalysts. Nonetheless, fabricating high-efficiency Ru-based catalysts with optimal atomic configurations continues to present a formidable challenge.

In recent times, single-atom catalysts (SACs) have garnered significant attention, especially precious metal SACs, due to their exceptional atomic utilization and uniform active site structure, making them highly attractive for deep oxidation reactions^15,16^. Studies further reveal that the architecture interfacial sites in SACs catalysts significantly influence both catalytic performance and thermal stability^17–20^. The complexity of soot oxidation occurring at the three-phase interface among solid catalysts, soot particles, and gaseous reaction gases (O_2_ and NO), adds to the challenge of designing and preparing efficient SACs catalysts^21^. This complexity demands careful consideration of both the intrinsic activity and stability of the catalyst for adsorbed/activated reactants, and the contact efficiency between soot particles and catalysts, as these factors play crucial roles in the reaction^22–27^. Compared to other oxides, Ceria (CeO_2_) proves to be an excellent cocatalyst, enhancing the catalytic performance and stability of precious metal active components in auto-exhaust catalysts because of its excellent oxygen storage/release properties^28–30^. In our previous works, we successfully constructed a series of Ce-based oxides as supports for the preparation of highly efficient supported noble metal soot oxidation catalysts^31^. Furthermore, our works highlight the importance of the strong interaction between Au NPs and CeO_2_ with the optimal crystal facet, which is crucial to adjust the intrinsic activity for O_2_ activation^32^. Despite these advancements, there remains limited research on SACs of single-atomic Ru anchored at the surface lattice of single-crystal CeO_2_, which holds the potential for replacing Pt/Pd-based catalysts in the field of soot purification.

In this study, we present a simple one-step synthesis of stabilized single-atom Ru sites confined on the surface lattice site of nanoflower-like CeO_2_ microspheres (Ru_1_/CeO_2_) using the gas bubbling-assisted membrane deposition (GBMD) method. These single-atom Ru active sites demonstrate remarkable thermal stability and enhance the activation efficiency of reactants (NO and O_2_). The Ru_1_/CeO_2_ catalyst, with Ru single sites on the CeO_2_ surface, exhibits excellent intrinsic catalytic performance and stability during soot oxidation under a loose contact model, outperforming both Ru nanoparticle and commercial Pt-based catalysts. Through comprehensive characterizations and density functional theory calculations, we identify the atomically dispersed Ru_1_O_5_ active site in the Ru_1_/CeO_2_ catalyst, along with the strong interface charge transfer within the Ru-O-Ce bond. The well-constructed active sites facilitate the formation of crucial surface NO_2_* intermediate species, which play a key role in the rate-determining step of NO oxidation to NO_2_, resulting in a significant reduction of the apparent activation energy during catalytic soot oxidation. This work emphasizes the advantages of synthesizing well-defined catalytic single sites on nanocrystals, and the combination of in-situ DRIFTS and DFT calculations provides valuable insights into the reaction mechanism. Furthermore, it establishes a methodological foundation for obtaining high-efficiency catalysts for auto-exhaust purification. The single-atom Ru catalyst offers a highly promising and cost-effective solution for auto-exhaust treatment systems.

## Results

### Chemical structure characterizations of Ru_1_/CeO_2_ and Ru_n_/CeO_2_ catalysts

**Fig. 1:**
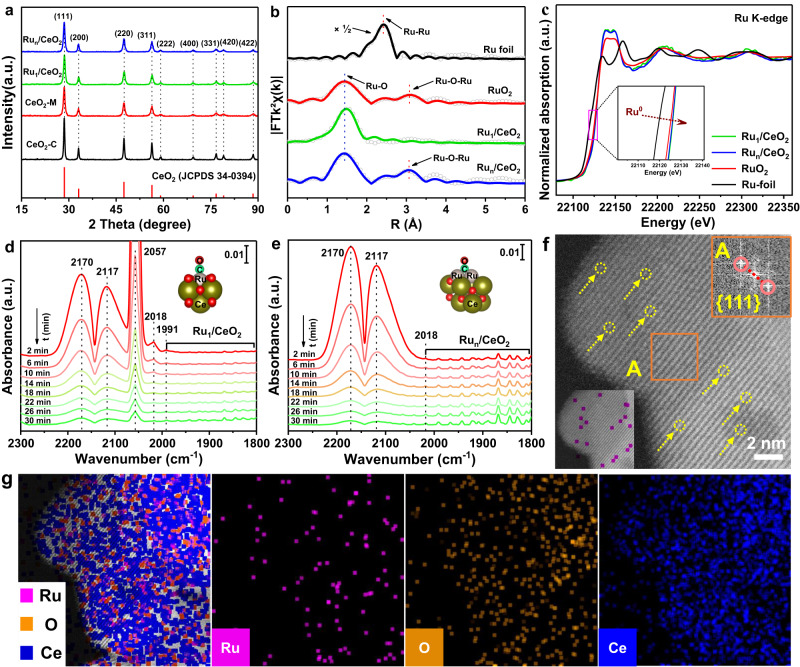
Structure characterization of Ru_1_/CeO_2_ and Ru_n_/CeO_2_ catalysts. **a** XRD patterns of all samples and standard card of CeO_2_. **b** EXAFS fitting results in R space and the Ru K- edge for Ru_1_/CeO_2_ and Ru_n_/CeO_2_ catalysts with RuO_2_ and Ru foil. The shell radii (*R*) of Ru-O and Ru-O-Ru are marked. **c** The Ru K-edge XANES profiles. **d** and **e** In-situ DRIFT spectra of CO adsorption at 50 ^o^C for the Ru_1_/CeO_2_ and Ru_n_/CeO_2_ catalysts with the extension of purge time (N_2_ flow, 30 mL min^-1^). **f** STEM-ADF image of Ru_1_/CeO_2_ catalyst with scale bars of 2 nm. The yellow circles represent atomically dispersed Ru sites. **g** STEM EDX mapping of Ru_1_/CeO_2_ catalyst (Purple, Ru; Orange, O; Mazarine, Ce).

The detailed synthesized processes of nanoflower-like CeO_2_ microspheres and Ru_1_/CeO_2_ catalyst were described (Supplementary Fig. 1**)**. The atomically dispersed Ru_1_/CeO_2_ catalyst was synthesized using the GBMD method^33^, while the reference catalyst of supported Ru nanoparticles (NPs) on nanoflower-like CeO_2_ (Ru_n_/CeO_2_) was obtained through the gas bubbling-assisted membrane reduction method^34^. Inductively coupled plasma optical emission spectroscopy (ICP-OES) analysis revealed that the actual Ru loading amounts in Ru_1_/CeO_2_ and Ru_n_/CeO_2_ catalysts are 0.46 wt.% and 3.80 wt.%, respectively (Supplementary Table 1). The powder X-ray diffraction (XRD) patterns of all samples exhibit characteristic peaks of CeO_2_ nanocrystals with a cubic fluorite structure (JCPDS 34-0394). Notably, no characteristic diffraction peaks associated with Ru or RuO_x_ NPs are detected, implying that the Ru species are highly dispersed on the surface of CeO_2_ (Fig. 1a). The lattice constant of CeO_2_ in the Ru_1_/CeO_2_ catalyst slightly decreases to 5.4084 Å compared to pure CeO_2_ (5.4101 Å) (Supplementary Table 1). This decrease is attributed to the substitution of Ce-sites on the surface lattice by smaller cation radius Ru ions (Supplementary Fig. 2). The SEM and TEM images provide a detailed view of the monodispersed nanoflower-like CeO_2_ microspheres, composed of single-crystal nanosheets (Supplementary Figs. 3a–d and 4). The thickness of these nanosheets is about 18 nm (Supplementary Fig. 5a, b), and the distance between two Ce atoms measures 3.1 Å, with clear step sites observed at the edge of the nanosheets (Supplementary Fig. 6).

In the N_2_ adsorption-desorption isotherms, all catalysts exhibit an H4 hysteresis loop in the P/P_0_ range 0.4-1.0, indicating the presence of a porous structure in the as-prepared samples (Supplementary Fig. 7a). The pore size distribution profiles of nanoflower-like CeO_2_, Ru_1_/CeO_2_, and Ru_n_/CeO_2_ catalysts all exhibit two peaks centered at 4.5 and 13.0 nm, in contrast to the single peak observed for CeO_2_-C at 8.0 nm (Supplementary Fig. 7b). The formation of these pores is attributed to the interwoven CeO_2_ nanosheets and stacked CeO_2_ nanoparticles (Supplementary Fig. 8a, b). To investigate the local electronic structure and coordination environment of Ru in the Ru/CeO_2_ catalysts, X-ray absorption measurements were conducted. The extended X-ray absorption fine structure (EXAFS) of Ru_1_/CeO_2_ shows only a primary peak at 1.49 Å, while the Ru_n_/CeO_2_ catalyst exhibits two peaks at 1.49 and 3.07 Å (Fig. 1b), corresponding to the Ru-O and Ru-O-Ru bond, respectively^35,36^. Further EXAFS curves fitting analyses provide insights into the coordination environment of Ru species in Ru_1_/CeO_2_ and Ru_n_/CeO_2_ catalysts, giving the coordination number of nearest-neighbor O atoms surrounding the isolated Ru atom (Fig. 1b and Supplementary Table 2). The coordination number of O atoms surrounding Ru atom in Ru_1_/CeO_2_ catalyst is 5.3, with a mean bond length of 2.01 Å, while the value of Ru_n_/CeO_2_ catalyst is 3.4, with the bond length of 2.04 Å. As a result, the atomically dispersed Ru species in the Ru_1_/CeO_2_ catalyst diffuse into the surface lattice of CeO_2_, forming coordination unsaturated Ru_1_O_5_ active sites. The atomic dispersion of Ru species in the Ru_1_/CeO_2_ catalyst is further corroborated by subsequent characterizations.

The X-ray photoelectron spectroscopy (XPS) spectra of Ru_1_/CeO_2_ and Ru_n_/CeO_2_ catalysts were acquired to determine the status of Ru species. The Ru 3*p* spin-orbit splits into 3*p*_3/2_ and 3*p*_1/2_ components, corresponding to the binding energy (BE) of 462.2 and 484.1 eV^37,38^. The two BE pairs (461.7 and 484.7 eV; 463.3 and 485.3 eV) corresponding to Ru 3*p*_3/2_ and 3*p*_1/2_ can be assigned to Ru^0^ and Ru^n+^ species, respectively (Supplementary Fig. 9)^39,40^. In the Ru_1_/CeO_2_ catalyst, the Ru 3*p* XPS only shows the oxidation state, indicating the presence of single positively charged Ru atoms. Conversely, the Ru 3*p* peak of the Ru_n_/CeO_2_ catalyst exhibits the coexistence of Ru^0^ and Ru^n+^ (4 ≤ *n* ≤ 6) species, attributed to the formation of a core-shell structured Ru@RuO_x_ layer in Ru sub-nanometric particles (SNPs)^41^. This suggests that the Ru species in the Ru_1_/CeO_2_ catalyst consist of single-atom Ru with a positively charged feature, while the Ru species in Ru_n_/CeO_2_ catalyst contain a certain amount of metallic state. Furthermore, the Ru K-edge adsorption position of the near-edge X-ray absorption fine structure (NEXAFS) spectra over the Ru_1_/CeO_2_ catalyst is slightly higher than that of Ru_n_/CeO_2_ and RuO_2_ observed through local magnification (Fig. 1c). This observation indicates that the valence state of single-atom Ru species in the Ru_1_/CeO_2_ catalyst is higher than +4, consistent with the result obtained from the Ru 3*p* XPS analysis.

Diffuse reflectance infrared Fourier transform spectrum of CO adsorption (CO-DRIFTS) on noble metal catalysts is widely used to investigate the atomic and electronic structures of metal-support binding sites. In the case of Ru/CeO_2_ catalysts, CO-DRIFTS can differentiate between active Ru sites in single atoms and nanoparticles. The CO-DRIFTS spectrum of Ru_1_/CeO_2_ catalyst shows four strong adsorption peaks (Fig. 1d), whereas the spectrum of Ru_n_/CeO_2_ catalyst shows only two strong adsorption peaks (Fig. 1e). The strong adsorption peaks centered at 2170 and 2117 cm^-1^ are assigned to the R and P branches of the rotation vibrational spectra of gas-phase CO species^42^. The strongest peak centered at 2057 cm^-1^ is ascribed to the C-O stretching vibration of dicarbonyl CO species (Ru^n+^(CO)_2_) adsorption on single atomically dispersed Ru^n+^ sites^43–45^. The peak centered at ~2018 cm^-1^ can be assigned to C-O vibration of CO linearly bound Ru sites with high coordination^46^, while the peak at ~1991 cm-^1^ could be ascribed to the CO adsorbed on oxygen vacancies or Ru-doped CeO_2_^47^. These findings indicate that the Ru species in Ru_1_/CeO_2_ catalyst exist as a single atomically dispersed ionic state, forming a Ru-O-Ce bond originating from the surface lattice site of CeO_2_, which has been substituted by a single Ru atom. On the other hand, the CO adsorption peak of the Ru_n_/CeO_2_ catalyst, centered at 2057 cm^-1^, is difficult to detect, but it demonstrates two weak bands at 1830 and 1860 cm^−1^, which can be attributed to the bridged adsorption of CO on two and three Ru atoms^46,48^, indicating the presence of Ru sub-nanometric particles (SNPs). With increasing purging time, the intensity of CO adsorption band on single atomic Ru sites decreases significantly, whereas that on Ru SNPs remains relatively unchanged. This suggests that the binding strength of CO to Ru_1_ is lower than that of Ru SNPs.

To further visualize the existence and dispersion state of the Ru species, aberration-corrected STEM images of the catalysts were obtained. Upon the introduction of the Ru species, Ru_1_/CeO_2_ catalyst maintains the initial nanoflower-like morphology of CeO_2_ (Supplementary Fig. 10a, b). The atomically dispersed Ru species in Ru_1_/CeO_2_ catalyst are faintly observed and coincided with the atomic lattice of CeO_2_ (Fig. 1f). The interplanar crystal spacing of CeO_2_ paralleled to the edge is 3.1 Å, as determined from the fast Fourier transform (FFT) pattern in the inset (A) of Fig. 1f, and corresponds to the exposed CeO_2_ (111) crystal facets. STEM images and corresponding Energy-dispersive X-ray (EDX) element mapping of the Ru_1_/CeO_2_ catalyst demonstrate the homogeneous dispersion of single-atom Ru species on the surface of CeO_2_ support (Fig. 1g). In contrast, the Ru species in Ru_n_/CeO_2_ catalyst exhibit a certain degree of aggregation and form small Ru SNPs (Supplementary Fig. 11a, b). The size distribution and EDX element mapping analysis in STEM-ADF images show the uniformed dispersion of Ru SNPs (*d* = 0.9 ± 0.2 nm) deposited on the surface of CeO_2_ nanosheets (Supplementary Fig. 12). Based on the results of EXAFS, in-situ CO-DRIFTS and STEM characterizations, it is evident that the state of Ru species in Ru_1_/CeO_2_ catalyst is predominantly isolated single-atom dispersion, occupying the surface lattice sites of CeO_2_ nanocrystals to form Ru_1_O_5_ structure. Conversely, the state of Ru species in the Ru_n_/CeO_2_ catalyst is RuO_2_ SNPs.

### Catalytic performance and kinetics of Ru/CeO_2_ catalysts in soot oxidation

**Fig. 2:**
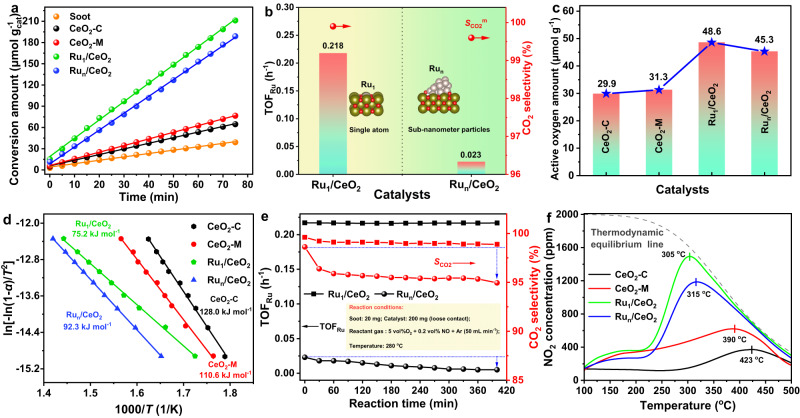
Catalytic performances of Ru_1_/CeO_2_ and Ru_n_/CeO_2_ catalysts in soot oxidation. **a** Soot conversion amount as a function of times by isothermal oxidation reaction at 280 ^o^C. **b** TOF_Ru_ over Ru_1_/CeO_2_ and Ru_n_/CeO_2_ catalysts. **c** Active oxygen amount by isothermal anaerobic titration at 280 ^o^C. **d** Ozawa plots of ln[-ln(1-*α*)/*T*^2^] vs *T*^-1^ for different soot conversion. **e** Time-TOF_Ru_ and the selectivity of CO_2_ over Ru_1_/CeO_2_ and Ru_n_/CeO_2_ catalysts (Reaction condition: Soot, 20 mg; Catalyst, 200 mg; Reactant gas flow, 50 mL min^-1^; At 280 ^o^C). **f** NO_2_ concentration curves of NO temperature-programmed oxidation.

The performance of all catalysts for soot oxidation was evaluated under the loose contact mode. To highlight the catalytic performance of the CeO_2_-M nanosheet catalyst, conventional powder-type CeO_2_ (CeO_2_-C) was synthesized as the reference sample. The relative reaction rates (*R*) of catalyzing soot oxidation were calculated through an isothermal oxidation reaction at 280 ^o^C (Fig. 2a**)**. The CeO_2_-M catalyst exhibits a higher *R* value (0.94 μmol g^−1^ min^−1^) compared to the CeO_2_-C sample (0.79 μmol g^−1^ min^−1^), indicating that the nanoflower-like morphology effectively enhances catalytic performance for soot oxidation. Additionally, the tight soot-catalyst contact mode over the same catalyst showed higher performance compared with the loose contact mode, which approximates real conditions (Supplementary Fig. 13a, b). Upon the introduction of Ru species, the *R* values of Ru_1_/CeO_2_ and Ru_n_/CeO_2_ catalysts remarkably increased to 2.59 and 2.38 μmol g^-1^ min^-1^, respectively. The actual Ru content obtained by ICP-OES allowed the representation of the reaction rate per each Ru atom, represented as the turnover frequency (TOF_Ru_). The TOF_Ru_ value of Ru_1_/CeO_2_ catalyst (0.218 h^-1^) is approximately nine-fold higher than that of Ru_n_/CeO_2_ catalyst (0.023 h^-1^) (Fig. 2b). This suggests that a single atomic Ru site possesses both high activity and atom utilization. Moreover, the selectivity of CO_2_ product (*S*_CO2_) over the Ru_1_/CeO_2_ catalyst is close to 100 %, allowing for the rapid removal of CO product emitted from vehicle exhaust. Furthermore, the Ru_1_/CeO_2_ catalyst exhibits excellent intrinsic catalytic performance during auto-exhaust soot oxidation compared to the commercial Pt-based catalysts (Supplementary Table 3).

The amount of active surface oxygen species plays a crucial role in catalytic performance for deep oxidation reactions, and it was determined by isothermal anaerobic titration at 280 ^o^C (Supplementary Fig. 14a-d). The presence of single atomic Ru species in the Ru_1_/CeO_2_ catalyst significantly increased the active oxygen amounts from 31.3 (CeO_2_-M) to 48.6 μmol g^-1^ (Ru_1_/CeO_2_), owing to the contribution of interface oxygen species in Ru-O-Ce bond (Fig. 2c and Supplementary Fig. 14a-d). To explore the reaction energy barrier on single atomic Ru sites during soot oxidation, the Arrhenius plots of the catalysts were analyzed (Fig. 2d). The Ru_1_/CeO_2_ catalyst exhibited the lowest apparent activation energy (*E*_a_, 75.2 kJ mol^−1^) in comparison with CeO_2_ and Ru_n_/CeO_2_ catalysts (Supplementary Table 4), indicating that single atomic Ru sites play a pivotal role in boosting the catalyzing soot oxidation.

Addressing the stability concerns associated with single-atom catalysts, we further investigated the stability of Ru species in the catalysts via the TOF_Ru_ and *S*_CO2_ values versus time (Fig. 2e). The TOF_Ru_ and *S*_CO2_ values of the Ru_1_/CeO_2_ catalyst remain relatively stable during 400 min, whereas those of the Ru_n_/CeO_2_ catalyst gradually decrease. In line with the cyclic test results of soot oxidation (Supplementary Figs. 15 and 16a, b), this confirms that the Ru_1_/CeO_2_ catalyst demonstrates higher catalytic stability compared with the Ru_n_/CeO_2_ catalyst. Additionally, the morphology and crystal phase structure remain unchanged during six-cycle TPO tests (Supplementary Fig. 17). On the other hand, ICP-OES analysis revealed a significant loss of Ru content on the used Ru_n_/CeO_2_ catalyst, which could be attributed to the volatilization of Ru species (Supplementary Fig. 18). The Ru_1_/CeO_2_ catalyst, with the surface lattice confinement single atom Ru, effectively inhibited the volatilization of Ru species. The CO-DRIFTS of the used Ru_1_/CeO_2_ catalyst demonstrated Ru species maintain a single atomically dispersed ionic state (Supplementary Fig. 19a, b), and STEM EDX mapping of Ru_1_/CeO_2_ catalyst also showed high dispersion of Ru species (Supplementary Fig. 20a, b). Conversely, the Ru species in the Ru_n_/CeO_2_ catalyst had aggregated into larger particles (Supplementary Fig. 21). The Ru 3*p* XPS spectra of the used Ru_1_/CeO_2_ catalyst still maintained the oxidation state with peaks located at 463.3 and 485.3 eV, corresponding to Ru^n+^ species (Supplementary Fig. 22). Raman spectra of the used Ru_1_/CeO_2_ revealed that the lattice-confined single-atom Ru_1_/CeO_2_ structure remains stable (Supplementary Fig. 23a, b). However, for the used Ru_n_/CeO_2_ catalyst, the peak intensity of RuO_2_ species (~323 cm^−1^) increased, indicating the aggregation of RuO_2_ into larger particles after six cycles of soot-TPO tests. After six cycles of tests, the I_D_/I_F2g_ value (area ratio of *D* peak to *F*_*2g*_ peak for Raman spectra) of the Ru_n_/CeO_2_ catalyst significantly decreased. This suggests that the interaction between the Ru species and CeO_2_ has changed, resulting in a decrease in the number of oxygen vacancies on the CeO_2_ surface. Additionally, the surface plasmon resonance (SPR) peak of UV-Vis red-shifted for the used Ru_n_/CeO_2_ catalyst (Supplementary Fig. 24), further indicating an increase in the average size of RuO_2_ nanoparticle during the reaction^49^. Consequently, the weakening of the interaction facilitates the volatility and migration of Ru species, leading to the formation of large particles, which in turn reduces the number of active sites and deactivates the catalyst during soot oxidation. These results demonstrate that the single-atom Ru sites anchored on the surface lattice of CeO_2_ nanocrystals exhibit high thermal and structural stability during soot oxidation.

Nitric oxide (NO) emissions from automobile exhaust are inevitable and can promote the removal efficiency of soot particles through NO oxidation into NO_2_, enhancing soot oxidation via a NO_2_-assisted mechanism^50^. We also investigated the role of NO over single-atom Ru active sites during catalytic soot oxidation (Supplementary Fig. 25a, b). Interestingly, the catalytic activity of Ru_n_/CeO_2_ catalyst is higher than that of the Ru_1_/CeO_2_ catalyst under the sole presence of O_2_. However, with the addition of NO, the *T*_50_ values significantly shift to the lower temperature for both Ru_1_/CeO_2_ and Ru_n_/CeO_2_ catalysts. The temperature difference (Δ*T*_50_) of Ru_1_/CeO_2_ and Ru_n_/CeO_2_ catalysts is 87 and 54 ^o^C under the sole presence of 5 vol% O_2_, and 5 vol% O_2_ + 0.2 vol% NO, respectively. This observation indicates that the presence of NO has a more pronounced effect on soot oxidation, emphasizing the critical role of NO activation and oxidation in enhancing catalytic activity for soot oxidation. It suggests that the Ru_1_/CeO_2_ catalyst can significantly promote the activation and oxidation of NO, thereby enhancing catalytic activity for soot oxidation via the NO_2_-assistant soot purification mechanism. The catalytic performance for NO oxidation was further evaluated using NO-temperature programmed oxidation (NO-TPO, Fig. 2f). It is noted that the Ru_1_/CeO_2_ catalyst has a higher NO_2_ concentration than the Ru_n_/CeO_2_ catalyst. Moreover, the Ru_1_/CeO_2_ (305 ^o^C) catalyst exhibits a lower temperature of NO_2_ concentration peak comparison with the Ru_n_/CeO_2_ catalyst (315 ^o^C). Therefore, the single-atom Ru sites anchored on the surface lattice of CeO_2_{111} facets exhibit improved catalytic performance for low-temperature NO oxidation compared with the Ru_n_/CeO_2_ catalyst. The relatively low temperature of NO oxidation over the single-atom Ru catalyst indicates that the Ru_1_/CeO_2_ catalyst demonstrates excellent activation and oxidation of NO molecules, contributing to its super-catalytic performance for NO_2_-assistant soot oxidation.

### Identifying active species and reaction pathways during soot oxidation

**Fig. 3:**
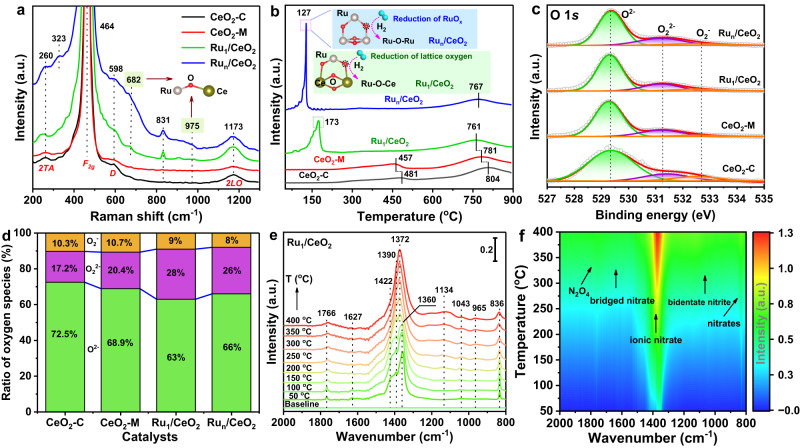
Identifying active species and reaction pathways of the catalysts during catalyzing soot oxidation. **a** Raman spectrum with an excitation wavelength of 532 nm. **b** H_2_-TPR profiles. **c** XPS spectra of O 1 *s*. **d** The ratio of oxygen species determined by XPS. **e** Temperature-dependent in-situ DRIFT spectra and (**f**) corresponding contour projection results of NO oxidation on Ru_1_/CeO_2_ catalyst (Catalyst, 10 mg; 5 vol% O_2_ and 0.2 vol% NO).

The performance of the catalysts during deep oxidation strongly depends on the presence of surface-active oxygen species, which are generated from activated O_2_ molecules by the low-coordinatively unsaturated cation (CUC) sites. Identifying the active species involved in catalyzing soot oxidation is crucial to understanding the reaction mechanism. We investigated the surface density of CUC sites induced by single-atom Ru species using Raman scattering spectra (Fig. 3a). The strong vibration peak at ~464 cm^−1^ corresponds to the first-order *F*_*2g*_ symmetry of CeO_2_ nanocrystals. Interestingly, the presence of Ru in the Ru_1_/CeO_2_ catalyst causes this peak to shifts down by ~7 cm^−1^ (Supplementary Fig. 26), suggesting that the atomically dispersed Ru species either lowers the symmetry of Ce-O bond on the Ru_1_/CeO_2_ catalysts or facilitates the transfer of electrons from Ru species to CeO_2_, affecting the electronic structure of the catalyst^51,52^. Additionally, the Raman peak centered at ~598 cm^−1^ corresponds to the defect-induced (*D*) mode of CeO_2_, and its intensity noticeably increases with the introduction of Ru species^53^. For Ru_1_/CeO_2_ and Ru_n_/CeO_2_ catalysts, two exclusive peaks at ~682 and ~975 cm^−1^ indicate the formation of the Ru-O-Ce bond, further confirming the presence state of Ru^n+^ species in the catalysts^54^. Moreover, a weak peak at ~323 cm^−1^ can be attributed to RuO_2_ formation in Ru_n_/CeO_2_ catalyst^55^. Furthermore, the peak at ~831 cm^−1^ is assigned to isolated two-electron surface defect sites on oxidized surfaces of Ce-based oxide^56^. To evaluate the surface density of oxygen vacancies, we examined the area ratio of *D* peak to *F*_*2g*_ peak (denoted as I_D_*/*I_F2g_)^57^. The Ru_1_/CeO_2_ catalyst exhibits the highest I_D_/I_F2g_ value (Supplementary Fig. 27), indicating that the formation of the Ru-O-Ce bond induces electronic transfer from Ru to Ce^4+^, leading to two Ce^4+^ ions replaced by two Ce^3+^ ions for creating each oxygen vacancy. As a result, the surface density of CUC sites is increased.

The amount of active oxygen species generated by CUC sites can be evaluated using temperature-programmed reduction with H_2_ (H_2_-TPR). CeO_2_-M catalyst exhibits a lower reduction temperature (457 and 781 ^o^C) and higher H_2_ consumption (1.389 mmol g^-1^) compared to CeO_2_-C catalyst (Fig. 3b and Supplementary Fig. 28), indicating that the exposed CeO_2_{111} facets enhance the oxidation property of surface oxygen species. In the H_2_-TPR profile of the Ru_n_/CeO_2_ catalyst, the first peak located at 127 ^o^C is assigned to the reduction of RuO_x_. However, the reduction of Ru species in the Ru_1_/CeO_2_ catalyst occurs at a higher temperature, 173 ^o^C, indicating a stronger interaction between Ru species and the support compared to that in the Ru_n_/CeO_2_ catalyst^56^. The reduction temperature of Ru species in the Ru_1_/CeO_2_ catalyst is higher than in the Ru_n_/CeO_2_, indicating two different existing states of Ru species. The initial H_2_ consumption rate reflects the activity of low-temperature surface oxygen species, and for supported Ru catalysts, their values increase more than thirteen-fold compared to bare CeO_2_-M (Supplementary Fig. 29), which results in the relatively low ignition temperature (*T*_10_) during catalytic soot oxidation (Supplementary Fig. 13). XPS was used to investigate the surface element state in the catalysts. The O 1 *s* XPS can be deconvoluted into three peaks (Fig. 3c). Surface peroxy-(O_2_^2-^) and super-oxygen (O_2_^-^) species, activated by CUC sites, are considered active oxygen species rather than lattice oxygen (O^2-^) during deep oxidation reactions. The percentage of active oxygen species in the single-atom Ru catalyst is the highest (37%) (Fig. 3d), suggesting that the CUC sites induced by Ru species greatly enhance the adsorption-activation of O_2_ molecules, leading to the formation of active oxygen species. This finding is consistent with the analysis result of Ce 3*d* XPS which shows that the Ru_1_/CeO_2_ catalyst has the highest percent (27.6%) of coordinatively unsaturated Ce^3+^ species, resulting from the strong Ru-O-Ce electronic interaction (Supplementary Fig. 30 and Supplementary Table 5). This electronic interaction is crucial for boosting the catalytic activity of soot oxidation^56^.

The transformation pathways of surface-active species over the catalysts were studied using in-situ DRIFTS. At 50 ^o^C, a series of NO_x_-containing species is observed on surface of the Ru_1_/CeO_2_ catalyst after introduction of NO and O_2_ into the reactor (Fig. 3e). These species include N_2_O_4_ dimer (1766 cm^-1^), bridging nitrates (1627 cm^−1^), monodentate nitrites (1422 and 1360 cm^-1^), ionic nitrites (1390 cm^-1^) and nitrates (836 cm^-1^) (Supplementary Table 6). As the reaction temperature increases, the three characteristic peaks of ionic nitrites and monodentate nitrites gradually merge into one new peak (1372 cm^-1^), and its intensity increases significantly. At the same time, the peak of bridged nitrates gradually weakens and eventually disappears, while new peaks of anionic (1134 cm^−1^) and bidentate (1043 and 965 cm^-1^) nitrates appear (Fig. 3f). These changes suggest that the adsorbed NO_x_ species gradually transform into labile NO_3_^-^ intermediates, which subsequently decompose into NO_2_. The Ru_n_/CeO_2_ catalyst exhibits similar evolution processes of in-situ DRIFTS spectra for NO oxidation (Supplementary Fig. 31), and the Ru_1_/CeO_2_ catalyst exhibits the largest relative intensity of surface NO_3_^-^ at 200 ^o^C (Supplementary Fig. 32), indicating that single atomic Ru species significantly promote the transfer from surface active oxygen to NO_3_^-^ intermediate. Finally, the gaseous NO_2_ produced by surface nitrate decomposition boosts catalyzing soot oxidation in the NO_2_-assisted mechanism. The electron and energy evolution processes of surface-active intermediate over the catalysts were further investigated through DFT calculations in the following discussion.

### Insight into the mechanism of single atomic Ru catalyst for soot oxidation

To gain insight into NO_2_-assisted catalytic mechanism for soot oxidation over Ru catalysts, the reaction pathways were investigated by DFT calculations. Model active sites of single atom Ru_1_ and Ru_10_ SNPs were constructed on the surface of CeO_2_ (110) facets (Supplementary Fig. 33a-c). The charge density difference maps of Ru_1_/CeO_2_ and Ru_10_/CeO_2_ were used to investigate the strong interaction between Ru and CeO_2_, and the extra number of transferred electrons was calculated using Bader charge analyses. The results showed that the Ru_1_ model can donate 1.46 e^-^ to CeO_2_ support (Supplementary Fig. 34a-c), while Ru_10_ model transfers a total of 1.26 e^-^, indicating a stronger electronic interaction between Ru_1_ and CeO_2_, which boosts the formation of CUC sites compared to Ru_10_ SNPs (Supplementary Fig. 35a-c).

**Fig. 4:**
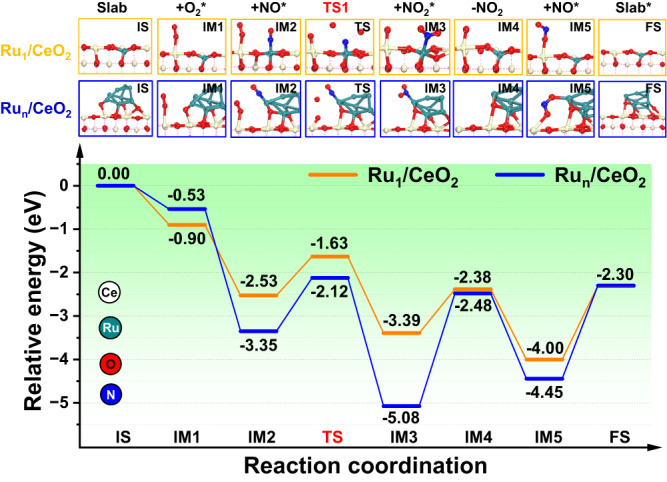
DFT calculations of Ru_1_/CeO_2_ and Ru_10_/CeO_2_ catalysts for NO oxidation. Reaction steps during catalyzing NO oxidation (Ru atom, black green; Ce atom, gray; O atom, red; N atom, blue). Color code: Ru_1_/CeO_2_ (brown line) and Ru_10_/CeO_2_ (blue line).

The reaction pathways and relative energy (*E*) during catalyzing NO oxidation can be divided into six stages as follows (Fig. 4): first, a gaseous O_2_ molecule adsorbs on the unsaturated coordination Ce site (Ce-O-Ru) over the catalyst surface through O-O-Ce bond to form surface adsorption O_2_* (IM1). The adsorption energy of O_2_ (*E*_ads_(O_2_)) is -0.9 and -0.53 eV for Ru_1_/CeO_2_ and Ru_10_/CeO_2_, respectively, indicating that the single Ru site easily adsorbs O_2_ molecule. The calculated charge density difference shows that the O_2_ molecule can be adsorbed on coordination unsaturated Ce atom of Ru_1_/CeO_2_ and Ru_10_/CeO_2_, with a total net charge transfer from Ru_1_/CeO_2_ surface to O_2_ being 0.56 e^-^ (Supplementary Fig. 36a, b) and 0.44 e^-^ for Ru_n_/CeO_2_ catalyst (Supplementary Fig. 37a, b). The adsorbed O_2_ molecule over Ru_1_/CeO_2_ catalyst gains the more electrons, facilitating the activation of O = O bond. Second, one NO molecule adsorbs on the Ru site of the catalyst surface through the O-N-Ru bond to form surface NO species (IM2). The NO adsorption energy (*E*_ads_(NO)) of Ru_10_/CeO_2_ (-2.82 eV) is lower than that of Ru_1_/CeO_2_ (-1.63 eV), and this strong adsorption capacity is not conducive to the subsequent reaction over Ru_10_/CeO_2_. Third, the O-O bond of the adsorbed O_2_ is activated to dissociate into atomic O* species, and adsorbed NO combines with O* to form the surface intermediate NO_2_* species (IM3). The reaction barriers for these steps are 0.9 and 1.23 eV with the corresponding transition states (TS1) of Ru_1_/CeO_2_ and Ru_10_/CeO_2_, respectively. Fourth, the NO_2_ species desorb from the catalyst surface (IM4). The NO_2_ desorption energy (*E*_des_(NO_2_)) for Ru_1_/CeO_2_ (1.01 eV) is lower than that of Ru_10_/CeO_2_ (2.60 eV), indicating that the desorption of NO_2_ molecules over the single atomic Ru site is easier than that of Ru_10_ site. This result in the rapid formation of NO_2_ on the surface of Ru_1_/CeO_2_ catalyst, a crucial step during soot oxidation. Fifthly, an additional NO molecule adsorbs on the O* to form ONO* (IM5). Finally, the formation of NO_2_* species desorbs from the catalyst surface, completing the reaction cycle (FS). The NO_2_ desorption energy (*E*_des_(NO_2_)) for Ru_1_/CeO_2_ (1.70 eV) is lower than that of Ru_10_/CeO_2_ (2.15 eV), indicating that the NO_2_ molecule was easily desorbed from the catalyst surface. Based on the above results, we found that NO molecules prefer to adsorb on Ru active sites on the surface of the catalysts, while O_2_ molecules are strongly adsorbed on the CUC sites. The rate-determining step of NO oxidation at the Ru-CeO_2_ interface is the formation of NO_2_* intermediate species. The stronger oxidizing NO_2_ can migrate to the soot surface along with the reaction gas flow and oxidize them to form CO_2_ via the indirect pathway (NO + 1/2O_2_ → NO_2_ and NO_2_ + soot → CO_2_). These DFT calculation results for NO oxidation are consistent with our experimental findings. The single active Ru site easily boosts NO oxidation to NO_2_ intermediate, which is beneficial to further promote soot oxidation.

## Discussion

We successfully fabricated stabilizing single-atom Ru sites on the surface lattice of nanoflower-like CeO_2_. The Ru_1_O_5_ coordination structure in the Ru_1_/CeO_2_ catalyst demonstrated remarkable thermal stability and activity in boosting the adsorption and activation of NO and O_2_ molecules, resulting in the formation of the crucial NO_2_ intermediate, which plays a key role in the NO_2_-assisted catalytic mechanism for soot oxidation. Therefore, the Ru_1_/CeO_2_ catalyst exhibits excellent intrinsic catalytic performance with a high turnover frequency (TOF_Ru_=0.218 h^−1^) and low apparent activation energy (*E*_a_ = 75.2 kJ mol^-1^) during auto-exhaust soot oxidation, surpassing both Ru nanoparticle (TOF_Ru_=0.023 h^-1^) and commercial Pt-based catalysts. Moreover, the single-atom Ru_1_/CeO_2_ catalyst demonstrates exceptional selectivity of the CO_2_ product (>99%) and remarkable durability during catalytic soot oxidation. This high-efficiency of the single-atom Ru catalyst offers a promising avenue for designing a considerably low-cost auto-exhaust treatment system, moving away from the reliance on costly Pt and Pd-based catalysts. The findings are also of significant importance for the further development of single-atom catalysts in the areas of deep oxidation and activated O_2_ reactions. The single-atom Ru catalyst paves the way for environmentally friendly and efficient exhaust treatment technologies, contributing to cleaner air and sustainable development.

## Methods

### Chemicals

Cerium (III) nitrate hexahydrate, acrylamide, and glucose were purchased from Aladdin Technology Co., Ltd. Standard ammonia solution (25 wt%) was purchased from Macklin Technology Co., Ltd. Ruthenium (III) chloride hydrate was purchased from J&K Scientific Ltd. Ethanol was purchased from Sigma Aldrich. All reagents and solvents were of analytical grade and used as received without additional purification.

### Catalysts preparation

#### Syntheses of nanoflower-like CeO_2_ microspheres and prepared conventional CeO_2_ nanoparticles

the nanoflower-like CeO_2_ microsphere was synthesized by the hydrothermal method^58^. The glucose (1.98 g, 0.010 mol) was dissolved into 80 mL of deionized water, and the addition of acrylamide (1.05 g, 0.015 mol) and hydrated cerium (III) nitrate (2.17 g, 0.005 mol) was stirred to form a transparent solution. Then, the standard ammonia solution (3.2 mL, 25 wt%) was dropwise to the above solution, and the solution became the sol with vigorous stirring at room temperature for 5 h. The color of the gelatinous mixture turned dark brown at the pH value of 10. Subsequently, the mixture was transferred into a 100 mL Teflon-lined autoclave and kept at 180 ^o^C for 72 h. After the natural cooling of the autoclave to room temperature, the sediment was separated by centrifugation, and the sample was washed with deionized water and ethanol three times. The nanoflower-like CeOHCO_3_ microsphere was obtained by drying at 80 ^o^C for 12 h, and was further two-steps calcined at 600 ^o^C for 4 h in N_2_ and at 500 ^o^C for 4 h in air. Finally, nanoflower-like CeO_2_ microsphere (denoted as CeO_2_-M) was obtained. Conventional CeO_2_ nanoparticles (CeO_2_-C) as reference sample was synthesized by the deposition-precipitation method. Cerium (III) nitrate hexahydrate (0.50 g) was dissolved into 20 mL of deionized water. Then, a standard ammonia solution (5 mL) was added dropwise under vigorous stirring. After aging for 30 min, the obtained product was washed with deionized water and ethanol. The product was dried at 80 ^o^C for 12 h and calcined at 500 ^o^C for 4 h in an air atmosphere to obtain general CeO_2_-C.

#### Syntheses of nanoflower-like Ru_1_/CeO_2_ and Ru_n_/CeO_2_ catalysts

The nanoflower-like CeO_2_-supported single-atom Ru (Ru_1_/CeO_2_) catalyst was synthesized by a gas bubbling-assisted membrane deposition (GBMD) method^33^. CeO_2_-M support (50 mg) was dispersed into deionized water (400 mL) with vigorous stirring, and 0.646 mL RuCl_3_·3H_2_O solution (0.01 g mL^−1^) was dropwise added into the above light-yellow slurry (denoted as Beaker I). A stabilizer (polyvinyl pyrrolidone, the molar ratio of Ru/PVP_unit_ is 1/100) was then transferred to Beaker I. A peristaltic pump with a rotation speed of 200 rpm was developed to form tubal cycling of the above solution mixture between the membrane reactor and Beaker I at a flow rate of 400 mL min^−1^. In the membrane reactor, the solution mixture flowed in the glass tube and outside the ceramic tubes. Prepared ammonia solution (1 mL standard ammonia solution was added to 40 mL deionized water) as a precipitant was immitted into the membrane reactor by a constant flow pump at a flow rate of 0.2 mL min^−1^. The ammonia solution infiltrated through the abundant holes on the walls of the two ceramic tubes into the glass tube, and the deposition of Ru ions occurred immediately until all the ammonia solution was completely consumed. The color of the slurry changed from light brown to dark gray, indicating that the Ru species were deposited. The mixture was filtered and washed with deionized water and ethanol three times, and the product was dried at 60 ^o^C for 12 h. Finally, the sample was calcined to obtain a single-atom Ru catalyst at 500 ^o^C for 4 h in air, denoted as Ru_1_/CeO_2_. The CeO_2_-M supported Ru nanoparticle (Ru_n_/CeO_2_) catalyst was prepared by using the gas bubbling-assisted membrane reduction method. This process is similar to GBMD, except that the high stoichiometric regents of RuCl_3_·3H_2_O (0.01 g mL^−1^, 5.167 mL) and PVP (Ru/PVP_unit_ is 1/100). Moreover, NaBH_4_ solution as a reductant (the molar ratio of NaBH_4_/Ru is 5/1) was immitted into the membrane reactor by a constant flow pump at a flow rate of 0.2 mL min^−1^. The NaBH_4_ solution (40 mL) infiltrated through the abundant holes on the walls of the two ceramic tubes into the glass tube, and the reduction of Ru ions occurred immediately until all the NaBH_4_ solution was completely consumed. The mixture was filtered and washed with deionized water and ethanol three times, and the product was dried at 60 ^o^C for 12 h. Finally, the sample was calcined at 500 ^o^C for 4 h in air, denoted as Ru_n_/CeO_2_.

### Characterizations

Powder X-ray (XRD) patterns were obtained by a diffractometer (Bruker D8 advance) using Cu-Kα radiation to obtain the phase structure of all as-prepared catalysts. The Ru K-edge X-ray absorption near edge structure (XANES) and extended X-ray absorption fine structure (EXAFS) experiments were carried out on the experiment assist system of SSRF and beamline BL13SSW and BL06B of the Shanghai Synchrotron Radiation Facility (SSRF). Raman spectra of all catalysts were measured on an inVia Reflex-Renishaw spectrometer with an excitation wavelength of 532 nm. Scanning electron microscopy (SEM), transmission electron microscope (TEM) and high-resolution transmission electron microscope (HRTEM) images were obtained by ZEISS Gemini SEM 300 and JEOL JEM LaB_6_ 2100, respectively. STEM-ADF images and EDX mapping were obtained by Hitachi HF5000, working at an accelerating voltage of 200 kV. The actual loading amounts of Ru in catalysts were determined by ICP-OES (Perkin Elmer, OPTIMA 7300 V). The pore size and specific surface area of catalysts were characterized by nitrogen adsorption−desorption experiments (Micromeritics TriStar-II 3020). Temperature-programmed reduction with H_2_ (H_2_-TPR) was carried out on a fixed-bed device. The surface element valence state was detected by X-ray photoelectron spectra (XPS, XPSPHI−1600 ESCA spectrometer). NO-TPO experiments were carried out on a fixed-bed tubular quartz reactor with flowed the gaseous contained O_2_ (5 vol%) and NO (0.2 vol%) balanced with N_2_ (50 mL min^−1^), and the products can be detected by online FT-IR. In situ diffuse infrared Fourier transforms spectra (in-situ DRIFTS) were carried on a Bruker FT-IR spectrometer (TENSOR II) equipped with a liquid nitrogen-cooling mercury-cadmium-telluride (MCT) detector. Before the in-situ CO-DRIFTS adsorption and desorption experiment, the sample (10 mg) was loaded into high-temperature IR cell with a ZnSe window (Pike Technologies), and pretreated in a 10 vol% H_2_ and balanced with N_2_ flow rate of 30 mL min^−1^ at 300 ^o^C for 30 min. After cooling down to 50 ^o^C under N_2_ flow (30 mL min^−1^) for 10 min, the background was collected. For the CO adsorption step, the CO (10 vol% CO in N_2_ balance) gas was fed into the cell at a flow rate of 30 mL min^−1^ for 30 min up to adsorption saturation. For the CO desorption, N_2_ flow (30 mL min^−1^) was purged into the cell to remove adsorbed CO, and the spectra were recorded at an apart of 2 min. In-situ DRIFTS of NO oxidation test, the catalyst (10 mg) was added into high-temperature IR cell with ZnSe window, and heated in N_2_ flow at 200 ^o^C for 30 min to remove adsorbed H_2_O and other materials. After cooling down to 50 ^o^C with N_2_ atmosphere, the background spectrum was recorded. Finally, the O_2_ (5 vol%), NO (0.2 vol%) and balanced with N_2_ were fed into cell at a flow rate of 50 mL min^−1^. IR spectra of the catalysts were recorded in a flow of 0.2 vol% NO/5 vol% O_2_/N_2_ balance (50 mL min^−1^) under heating from 50 to 400 ^o^C.

### Catalytic activity and kinetic tests

The catalytic activity for soot oxidation was evaluated by soot-TPO in a tubular quartz reactor using Printex-U as model soot particles. The loose contact was obtained by mixing the catalyst (100 mg) and soot (10 mg) with a spoon for 10 min, and the tight contact was obtained by grinding the above mixture for 10 min. The reaction temperature in soot-TPO rises from 150 to 550 ^o^C with a rising rate of 2 ^o^C min^−1^. The reaction gases were composed of 5 vol% O_2_ and 0.2 vol% NO balanced with Ar, and the total flow rate was 50 mL min^−1^ passed through the mixture. The gas product of CO_2_ and CO in the outlet gas was monitored by an online gas chromatograph (GC 9890B, Shanghai). The catalytic performance was evaluated by the TOF_Ru_, which can be defined by the ratio of the isothermal reaction rate (*R*) to the actual amount of Ru-supported in the catalysts. The temperature at 10%, 50%, and 90% of soot conversion from soot-TPO test denotes *T*_10_, *T*_50_, and *T*_90_, respectively. The selectivity of CO_2_ (*S*_CO2_) can be calculated by the ratio of CO_2_ concentration to the sum of CO and CO_2_ concentration, and the *S*_CO2_ was calculated by the following formula:

1 $${{{{{{\rm{S}}}}}}}_{CO2}(\%)=\frac{{[{{{{{{\rm{CO}}}}}}}_{2}]}_{{{\rm{out}}}}}{{[{{{{{\rm{CO}}}}}}]}_{{{\rm{out}}}}+{[{{{{{{\rm{CO}}}}}}}_{2}]}_{{{\rm{out}}}}}$$

Here, the [CO_2_]_out_ and [CO]_out_ represent the outlet CO_2_ and CO concentration (ppm), respectively. *S*_CO2_^m^ was defined as *S*_CO2_ with the maximum value of CO_2_ concentration.

The *R* values can be obtained by isothermal reaction at 280 ^o^C for soot oxidation, the conversion of soot remains basically unchanged and the conversion rate is less than 10%. The *R* values for all catalysts were calculated by the slopes of the soot conversion amount with time, which are reflected in the concentration of CO_2_ per unit of time. The soot conversion rate (*R*) was calculated as following^59^:

2 $${{{{{\rm{R}}}}}}(\upmu {{{\rm{mol}}}}{{{\rm{g}}}}^{-1}{\min }^{-1})=\frac{{{{{{\rm{QC}}}}}}}{22400\times {{{{m}}}}}$$

Where *Q* represents the gas flow rate (mL min^−1^), *C* represents the concentration of CO_2_ measured by isothermal reactions (ppm), and *m* represents the weight of the catalyst (g).

The active oxygen (*O*_a_) amount can be obtained by isothermal anaerobic titrations^31^. The TOF_Ru_ value of the Ru/CeO_2_ catalyst can be calculated by the following equation:

3 $${{{{{\mathrm{TOF}}}}}}_{{{{{\mathrm{Ru}}}}}}({{{{{\mathrm{h}}}}}}^{-1})=\frac{({R2}-{R1}){M}\times 6\times {10}^{-6}}{mw}$$

Here, the *R2 and R1* represent the isothermal reaction rate for Ru/CeO_2_ and CeO_2_ catalysts (μmol g^−1^ min^−1^), respectively. *M* is the atomic weight of Ru (101.07 g mol^−1^), m is the mass of the catalyst (g) and the *w* (wt.%) is the actual loading amount of Ru species on the surface of CeO_2_.

The apparent activation energy (*E*_a_) can be calculated by the Coats-Redfern interregnal method^60^. The *E*_a_ values are obtained by the following equation:

4 $${{{{\mathrm{ln}}}}}\left[-\frac{{{{{\mathrm{ln}}}}}(1-{{{{{\rm{\alpha }}}}}})}{{{{{{{\rm{T}}}}}}}^{2}}\right]=\,{{{{\mathrm{ln}}}}}\left[\frac{{{{{{\rm{AR}}}}}}}{{{{{{\rm{\beta }}}}}}{{{{{\rm{E}}}}}}a}\left(1-\frac{2{{{{{\rm{RT}}}}}}}{{{{{{\rm{E}}}}}}a}\right)\right]-\frac{{{{{{\rm{E}}}}}}a}{{{{{{\rm{RT}}}}}}}$$

Here, the *α* is the conversion value of soot, %. *T* is the reaction temperature, K. *A* is the pre-exponential factor, s^−1^. *E*_a_ is the apparent activation energy, kJ mol^−1^. *R* represents the ideal gas constant, 8.314 J mol^−1^ K^−1^. *β* represents the heating rate, K min^−1^.

The stability of catalysts was evaluated by Time-TOF_Ru_ and recycling soot-TPO tests. For the Time-TOF_Ru_ test, a spatula was used to mix soot (20 mg) and catalyst (200 mg) thoroughly to form loose contact. The mixture was heated on a fixed-bed tubular quartz microreactor with an inner diameter of 6 mm, and heated from 50 to 280 ^o^C at a heating rate of 2 ^o^C min^−1^ under a reactant gas flow (50 mL min^−1^) of O_2_ (5 vol%) and NO (0.2 vol%) balanced with Ar. The reaction temperature was kept at 280 ^o^C (lasting for 400 min) in an approximate kinetic regime because the conversion of soot oxidation was low (10%) and nearly constant over time. The outlet gas concentration of CO and CO_2_ is detected by an online gas chromatograph. When the temperature reaches 280 ^o^C, the outlet gas concentration is detected for the first time as the starting point. Based on the above process, the production CO_2_ concentration of Ru/CeO_2_ and CeO_2_ catalysts can be obtained at specific times, respectively. The CO_2_ produced by the bare Ru species can be defined as the concentration produced by Ru/CeO_2_ subtraction of the concentration of CO_2_ produced by the CeO_2_. The TOF_Ru_ value was calculated as follows:

5 $${{{{{\mathrm{TOF}}}}}}_{{{{{\mathrm{Ru}}}}}}({{{{{\mathrm{h}}}}}}^{-1})=\frac{{C}_{{{{{\rm{CO2}}}}}}^{p}{QM}\times 6\times {10}^{-5}}{22400\times {mw}}$$

Where the${{{{{{\rm{C}}}}}}}_{CO2}^{p}$ represents the produced CO_2_ concentration (ppm) by the bare Ru species, the value can be defined as the concentration produced by Ru/CeO_2_ subtraction of the concentration of CO_2_ produced by the support CeO_2_. *Q* represents the gas flow rate (mL min^−1^), *M* is the atomic weight of Ru (101.07 g mol^−1^), *m* is the mass of the catalyst (g) and the *w* (wt.%) is the actual loading amount of Ru species on the surface of CeO_2_. Finally, the profile of Time-TOF_Ru_ and the selectivity of CO_2_ over Ru_1_/CeO_2_ and Ru_n_/CeO_2_ catalysts were gained within 400 min.

For the cyclic stability test, the catalyst was recycled after the first test of soot-TPO. The detailed process is as follows: firstly, the catalyst was taken out from the reaction quartz tube and then the quartz cotton was cleaned off from the surface of the catalyst (this process loses about 2% of the catalyst mass). Each time, the used catalyst was mixed with 10 mg soot particles, and the next activity test (soot-TPO) was performed, the values of *T*_10_, *T*_50_, *T*_90_ and *S*_CO2_^m^ for Ru/CeO_2_ catalysts can be obtained. The above experimental process was repeated six times, and six groups of activity data were finally acquired, while no additional catalyst was added for each test during six cycles of soot-TPO.

### Density functional theory (DFT) calculations

DFT calculations were carried out by using the VASP package code^61^. The exchange-correlation energy function was described in the Pardew-Burke-Ernzerhof (PBE) generalized gradient approximation (GGA)^62^. The kinetic cutoff energy is 400 eV for the plane-wave basis set, and the *k* point was set to the *γ* point in the Brillouin zone. All calculations for CeO_2_ models were performed by the DFT + U method for the present study. The value of U_eff_ was set to 5 eV for Ce 4 *f* electrons. CeO_2_ supercells (2 x 4) were built, with (110) surfaces exposed. The bottom two atomic layers were fixed during all calculations. The thinness of the vacuum layer for all the models was set as 15 Å. Combined with the results of STEM-ADF and EXAFS characterizations, one of the Ce atoms in the surface layer was replaced by a Ru atom to simulate the single-atom Ru structure, and the Ru nanoparticles contain 10 atoms in the Ru_n_/CeO_2_ model^63^. The adsorption and desorption energy were calculated by the equation:

6 $${{E}}_{ads}={{E}}_{MS}-{{E}}_{S}-{{E}}_{M}$$

7 $${{E}}_{des}={{E}}_{S}+{{E}}_{M}-{{E}}_{MS}$$

Where *E*_ads_ is the adsorption energy, *E*_des_ is the desorption energy, *E*_MS_ is the total energy of a surface slab with the adsorbate, and *E*_S_ is the energy of pure substrate. *E*_M_ represents the energy of an adsorbate molecule.

The climbing image-nudged elastic band (CI-NEB) code was used to identify the reaction coordinates from IS to FS^64,65^, which located the transition state (TS). The activation energy (*E*a) and reaction energy (*E*_r_) were determined with the following equation:

8 $${{E}}_{a}={{E}}_{TS}-{{E}}_{FS}$$

9 $${{E}}_{r}={{E}}_{FS}-{{E}}_{IS}$$

Where *E*_IS_, *E*_TS_, and *E*_FS_ represent the energy of IS, TS, and FS, respectively.

### Supplementary information

Supplementary Information

### Source data

Source Data

## Data Availability

The data that support the findings of this study are included in the published article (and its Supplementary Information). Source data are provided with this manuscript and is available from the corresponding author upon request. Source data are provided with this paper.
