# Supplementary material for: A single site ruthenium catalyst for robust soot oxidation without platinum or palladium
Source: Nat Commun. 2023 Nov 6;14:7149. doi: 10.1038/s41467-023-42935-7 (PMC10628289; doi:10.1038/s41467-023-42935-7)
Supplement: Supplementary file 1 — Supplementary Information [file 41467_2023_42935_MOESM1_ESM.pdf]

Supplementary Information

A single site ruthenium catalyst for robust soot oxidation without platinum or palladium

Yuanfeng Li[1], Tian Qin[2], Yuechang Wei[1*], Jing Xiong[1], Peng Zhang[1], Kezhen Lai[1], Hongjie Chi[1], Xi Liu[2*], Liwei Chen[2], Xiaolin Yu[3*], Zhen Zhao[1*], Lina Li[4] and Jian Liu[1]

[1] State Key Laboratory of Heavy Oil Processing, Key Laboratory of Optical Detection Technology for Oil and Gas, China University of Petroleum, Beijing, 102249, P. R. China

[2] School of Chemistry and Chemical, In-situ Center for Physical Science, Shanghai Jiao Tong University, 200240, Shanghai, P. R. China

[3] State Key Laboratory for Structural Chemistry of Unstable and Stable Species, Beijing National Laboratory for Molecular Sciences (BNLMS), CAS Research/Education Center for Excellence in Molecular Sciences, Institute of Chemistry, Chinese Academy of Sciences, Beijing, 100190, China

[4] Shanghai Synchrotron Radiation Facility, Shanghai Advanced Research Institute, Shanghai, China

* Corresponding author: weiyc@cup.edu.cn; liuxi@sjtu.edu.cn; icecoolyu@iccas.ac.cn; zhenzhao@cup.edu.cn

Supplementary Figures

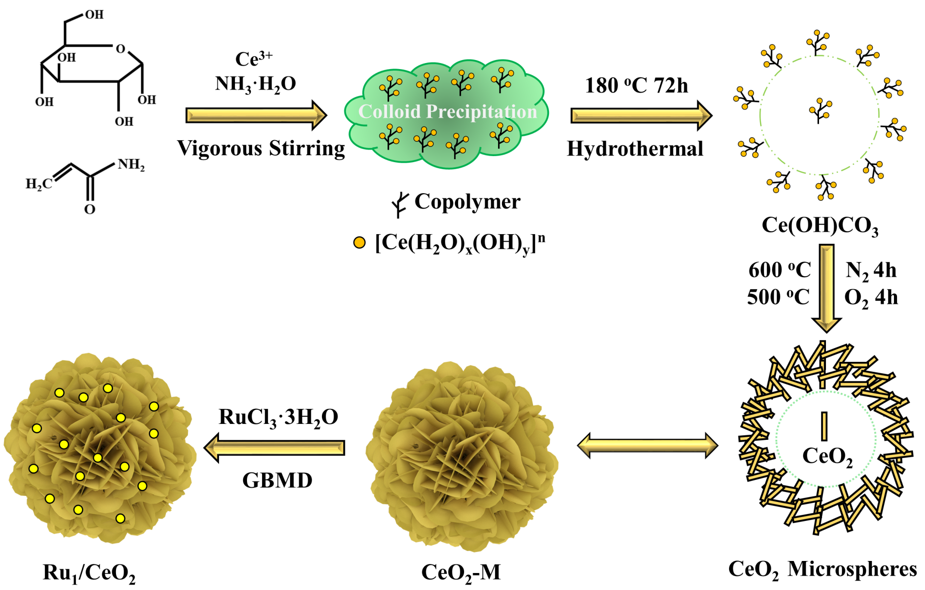

Supplementary Figure 1. A schematic diagram describing the synthesis of nanoflower-like Ru_1/CeO_2 microsphere catalysts.

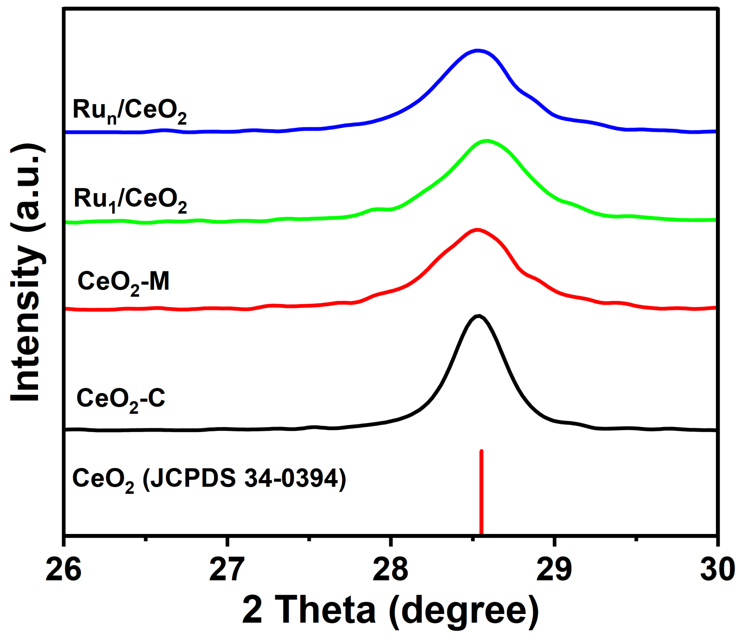

Supplementary Figure 2. XRD patterns of all as-prepared catalysts partially enlarged view of the diffraction peaks associated with (111) planes.

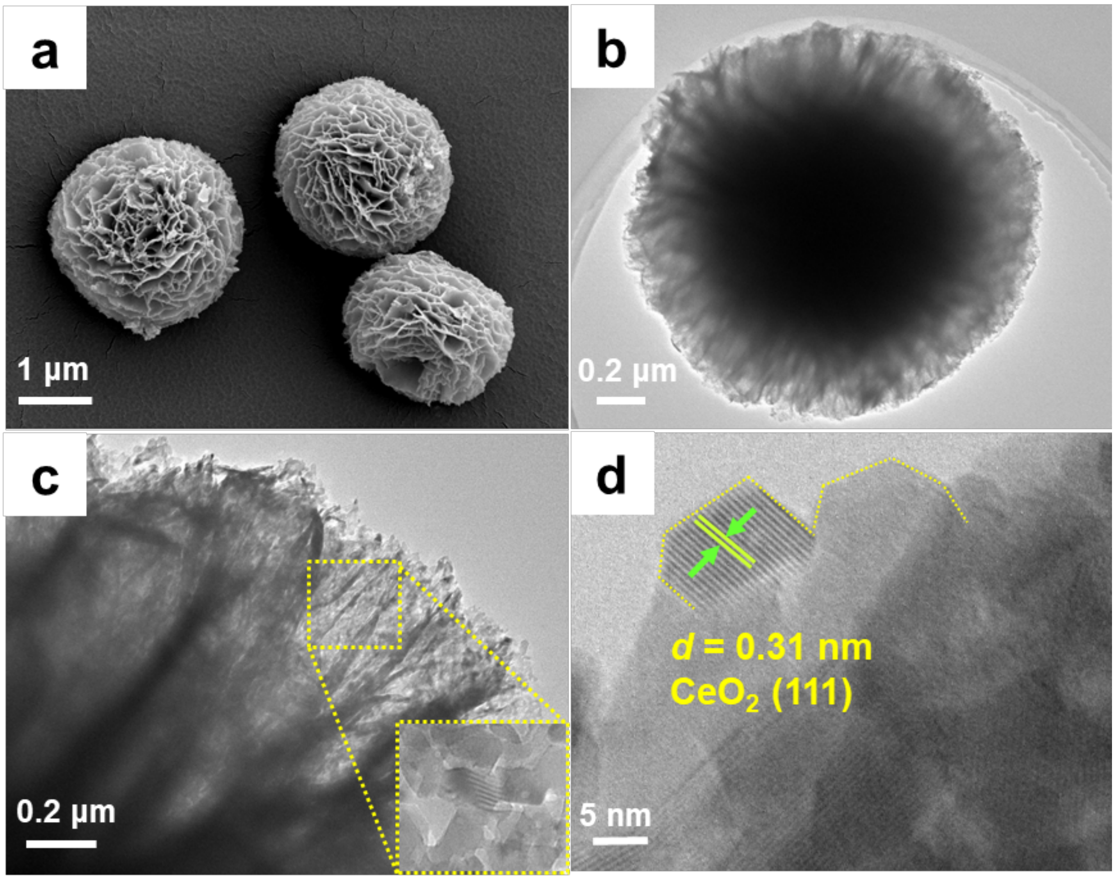

Supplementary Figure 3. SEM image (a), TEM (b, c) and HRTEM images (d) of nanoflower-like CeO_2 microsphere.

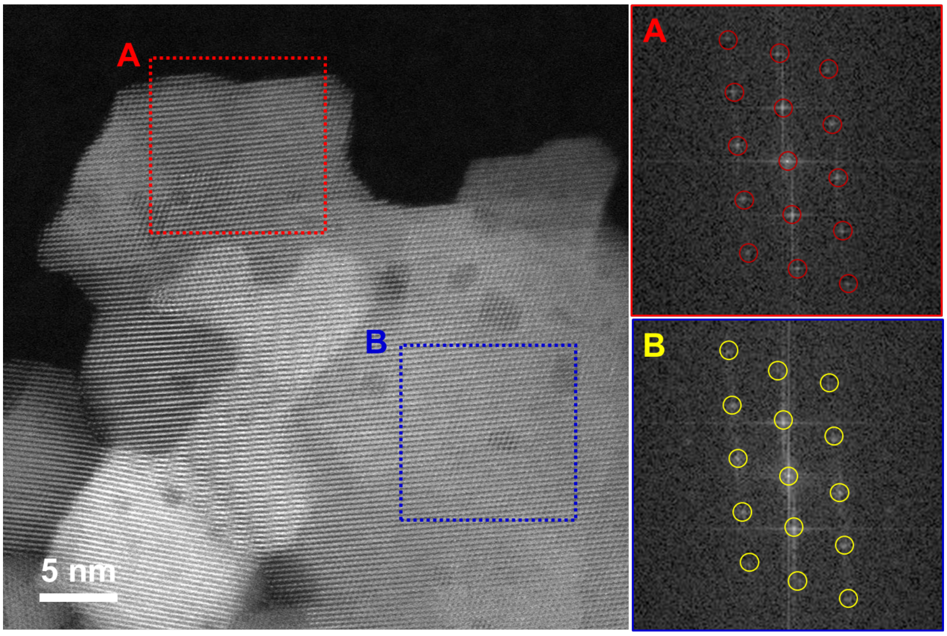

Supplementary Figure 4. STEM-ADF image of CeO_2-M catalyst with scale bars of 5 nm (A and B represent the two regions and corresponding FFT images).

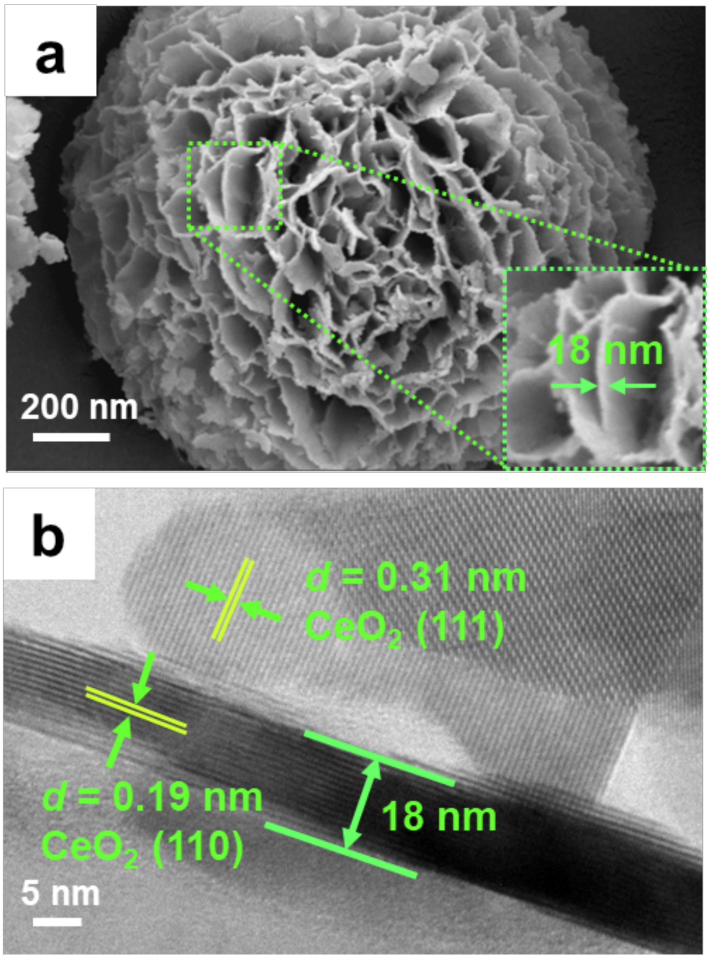

Supplementary Figure 5. SEM image (a) and HTEM image (b) of CeO_2-M catalyst.

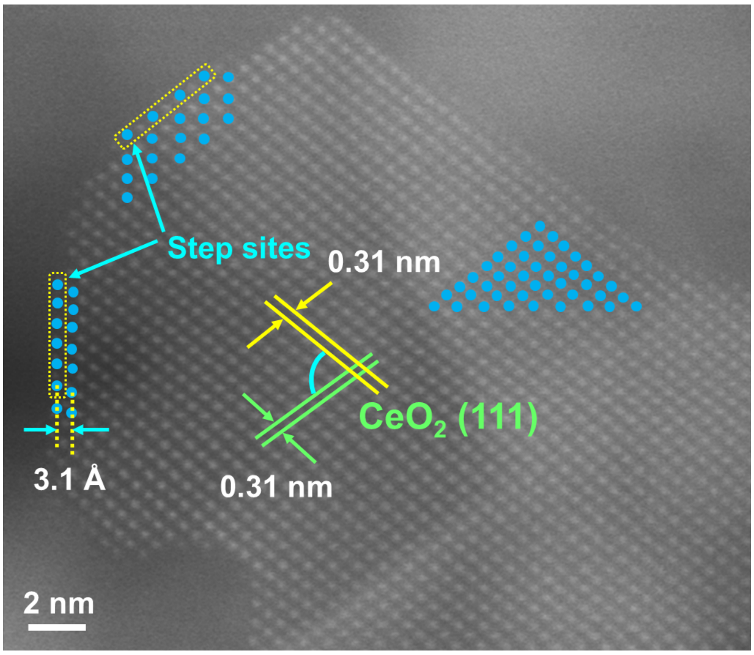

Supplementary Figure 6. STEM-ADF image of nanoflower-like CeO_2 microsphere with scale bars of 2 nm.

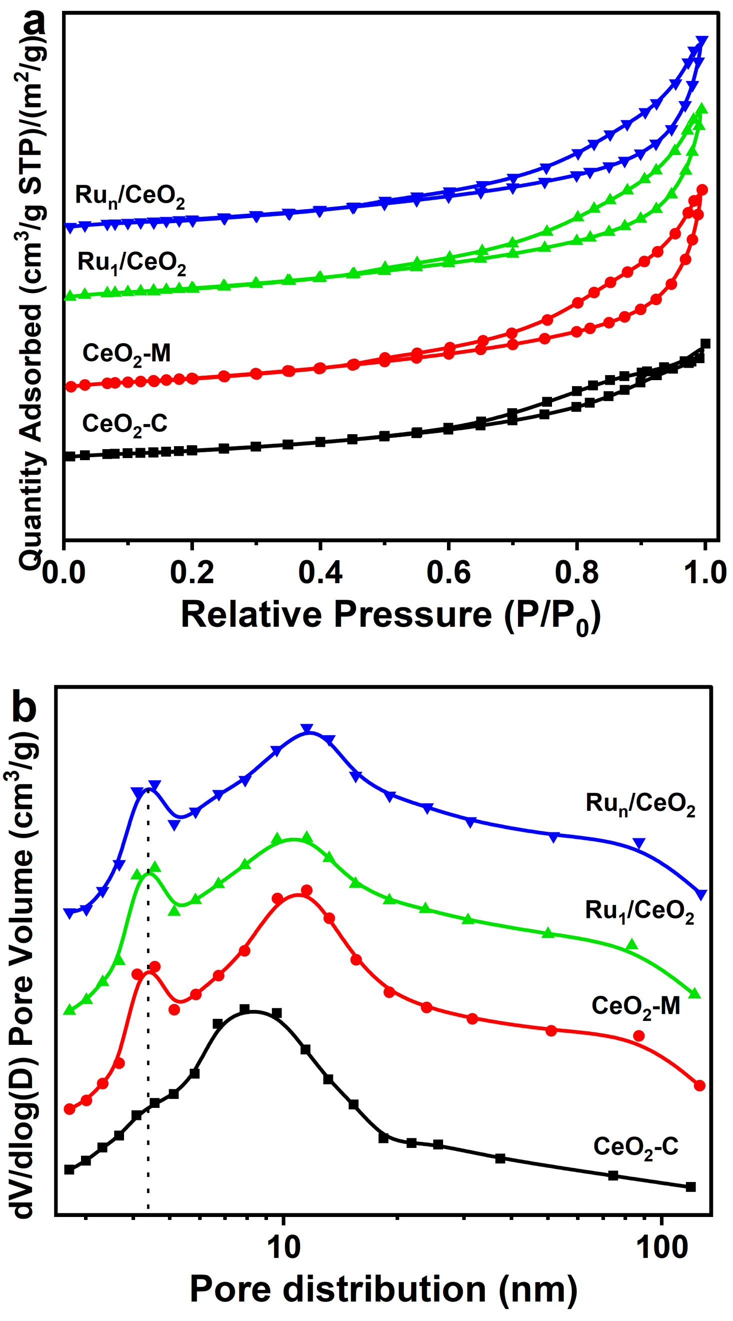

Supplementary Figure 7. N_2 adsorption-desorption isotherms (a) and pore size distribution curves (b) of CeO_2-C, CeO_2-M, Ru_1/CeO_2, and Ru_n/CeO_2 catalysts.

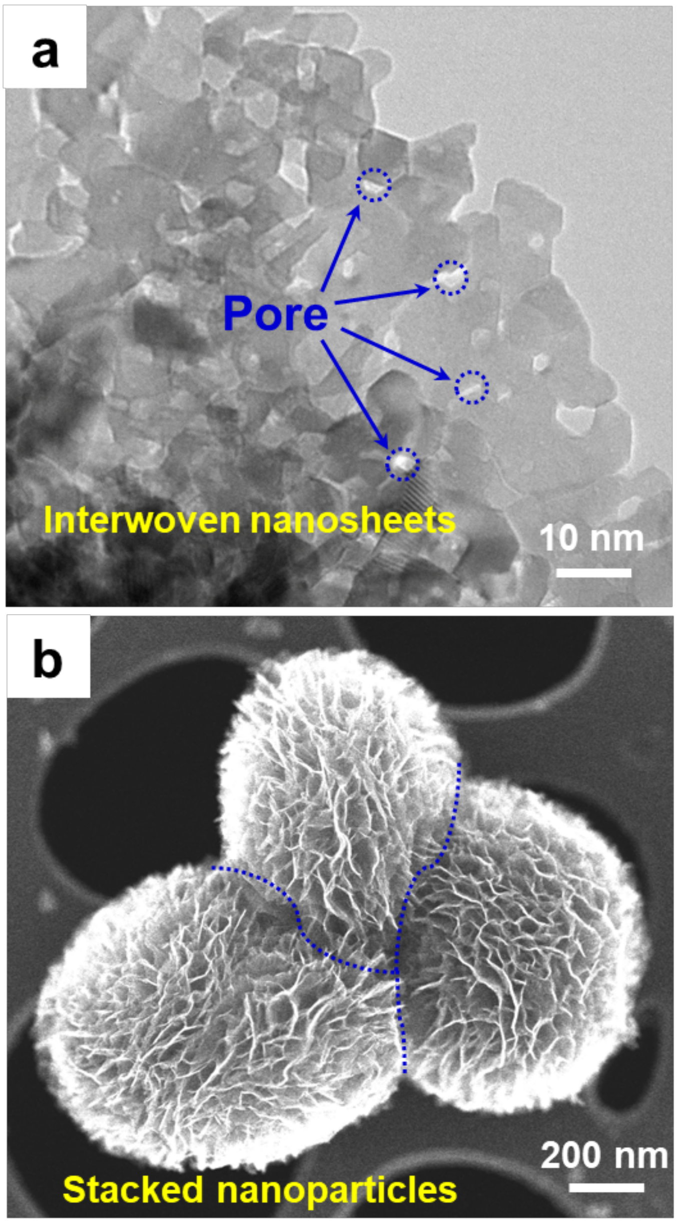

Supplementary Figure 8. TEM (a) and STEM-SE (b) images of CeO_2-M sample, the mesoporous and stacked porous in CeO_2-M highlighted by the blue circles and blue line.

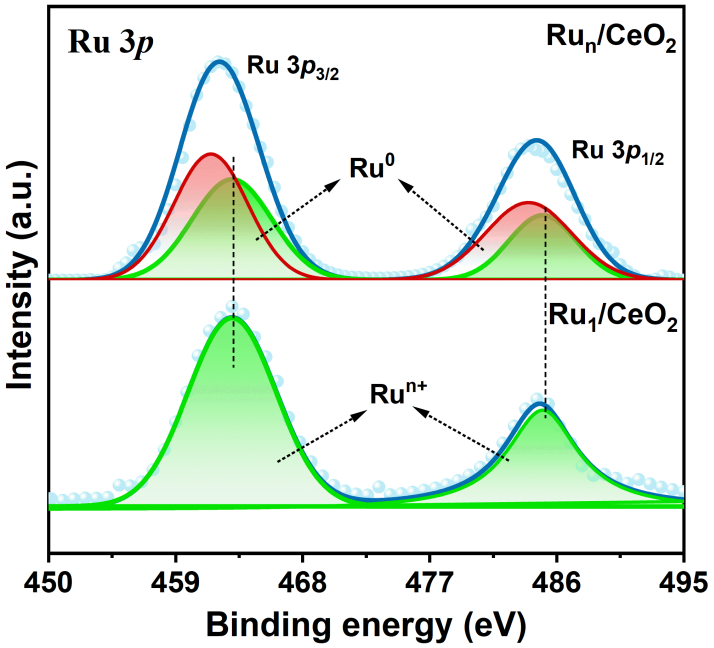

Supplementary Figure 9. XPS spectra of Ru 3*p* for Ru_1/CeO_2 and Ru_n/CeO_2 catalysts.

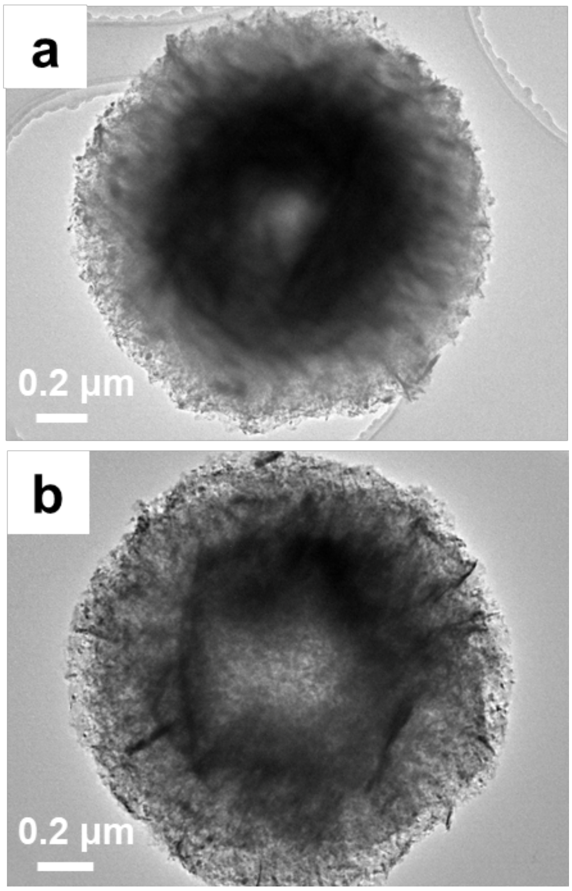

Supplementary Figure 10. TEM images of Ru_1/CeO_2 (a) and Ru_n/CeO_2 catalysts (b).

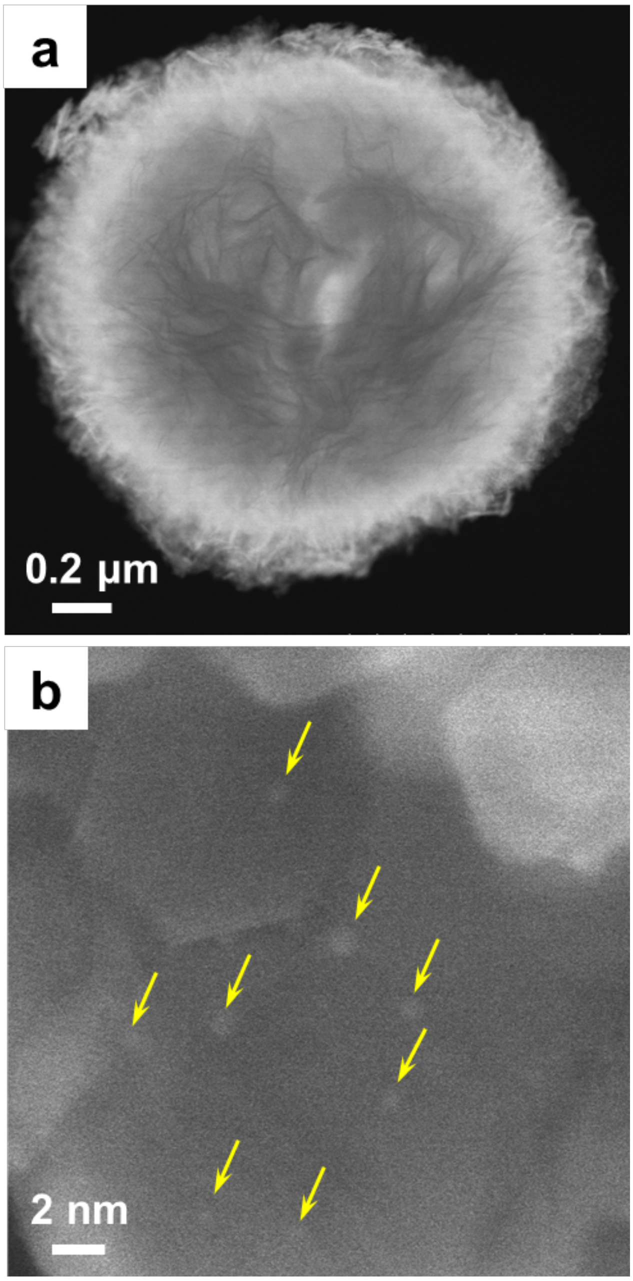

Supplementary Figure 11. (a) STEM-ADF and (b) STEM-SE images of Ru_n/CeO_2 catalyst (Sub-nanometric Ru particles are highlighted by the yellow arrow).

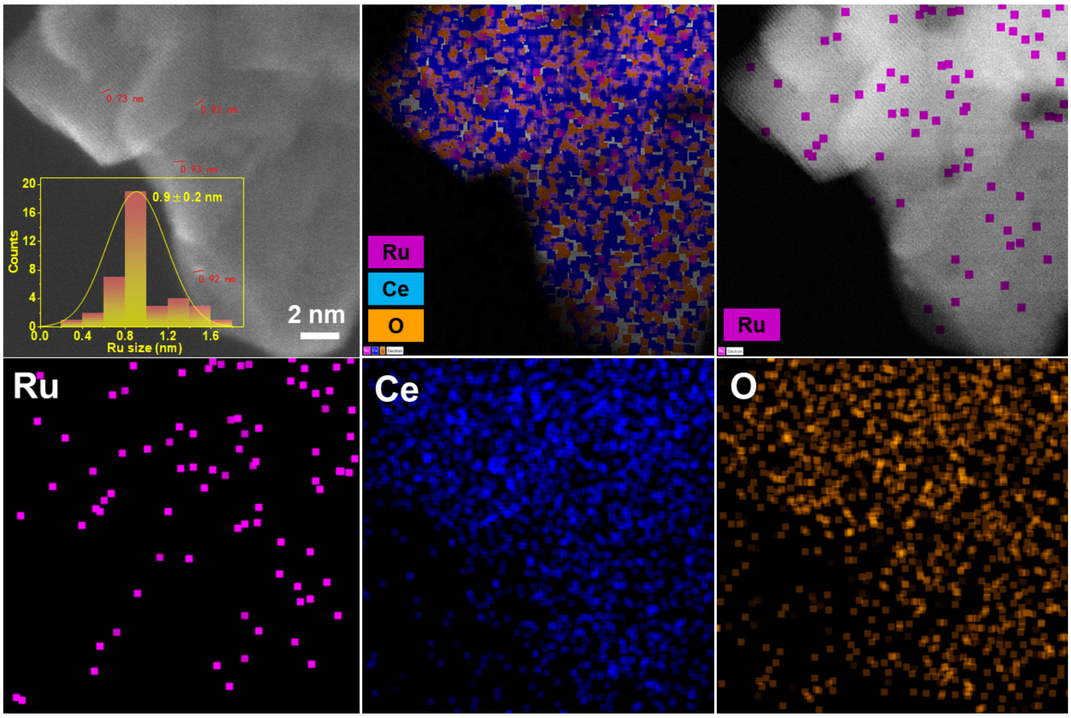

Supplementary Figure 12. STEM image with size distribution as inset and corresponding EDX mapping images of Ru_n/CeO_2 catalyst. In EDX-mapping images, purple dots represent the Ru element, blue dots represent the Ce element and brown dots represent the O element.

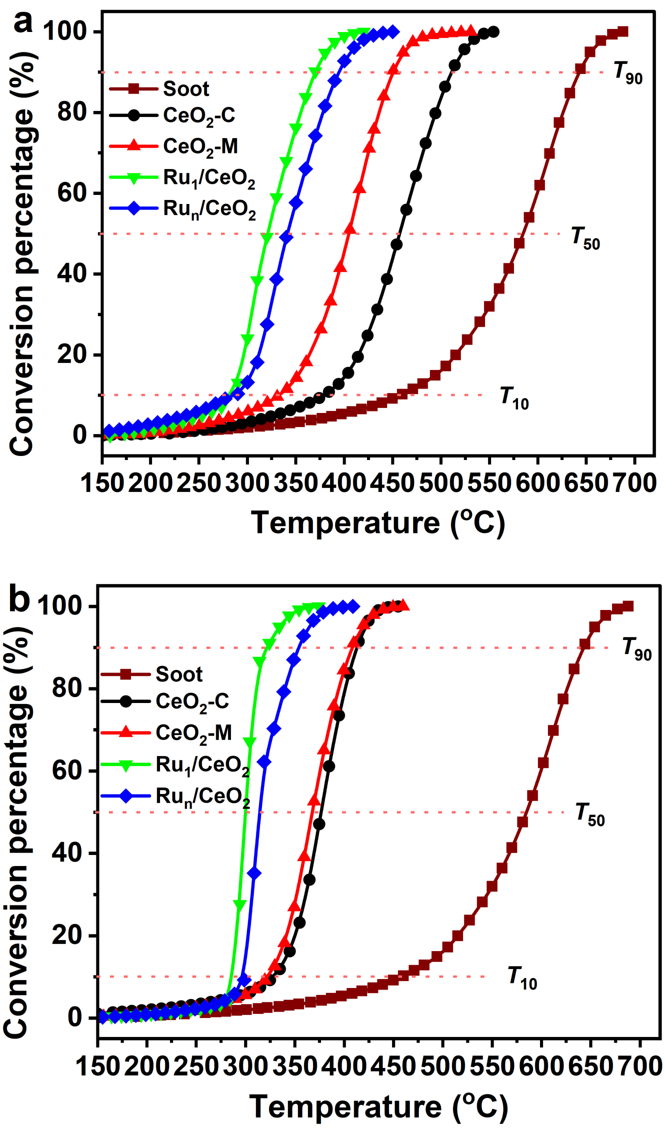

Supplementary Figure 13. Conversion percentage of soot oxidation over all as-prepared catalysts under different contact methods. (a) loose contact and (b) tight contact between soot particles and catalysts.

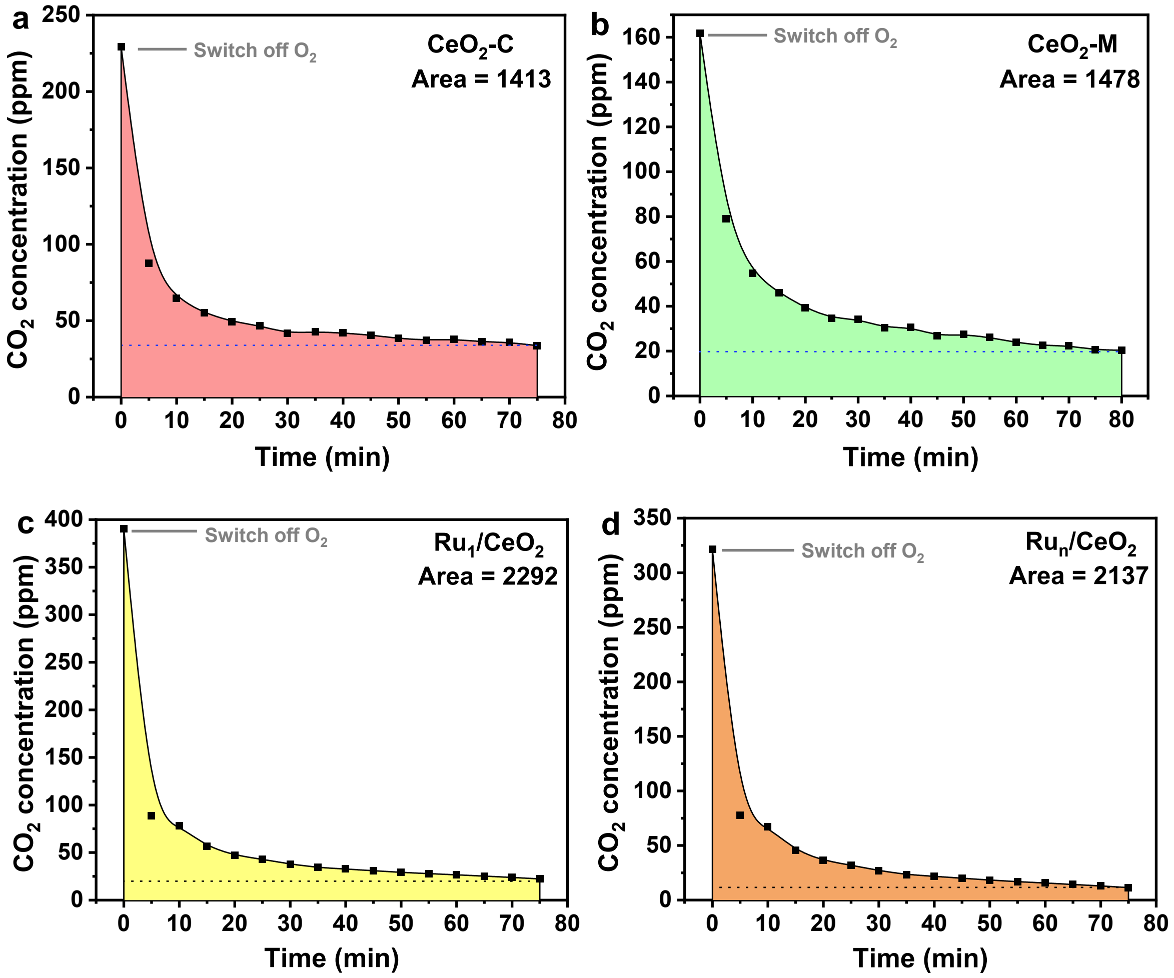

Supplementary Figure 14. CO_2 concentration curves at 280 ºC as a function time under the loose contact condition over the catalysts after O_2 is removed from the reactant feed. (a) CeO_2-C, (b) CeO_2-M, (c) Ru_1/CeO_2 and (d) Ru_n/CeO_2.

Note: For isothermal anaerobic titrations, the feed of O_2 was instantaneously removed and the transient decay of the CO_2 concentration from the steady state was monitored. Since no further oxygen was added to the reaction system, the amount of stored oxygen can be calculated from the subsequent total production of CO_2. The amount of active oxygen can be obtained by the following formula:

$$\mathrm{O*amount}(\mu\mathrm{mol\,g}^{-1}) = \frac{2\times10^{-6} P_0 V A}{R T m}$$

Here, P_0 is the atmospheric pressure, Pa; V is the volumetric flow rate, $m^3\ s^{-1}$; A is the integral of CO_2 concentration curves as a function of time during the isothermal anaerobic titration, s; R represents the gas constant; T represents the reaction temperature, K; and m represents the catalyst weight, g.

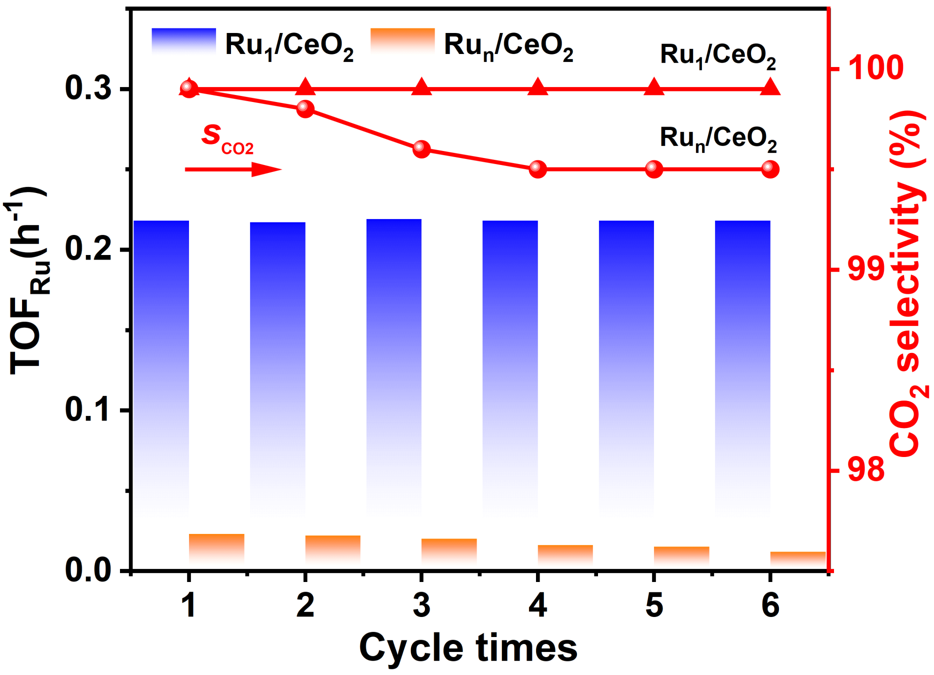

Supplementary Figure 15. Cyclic stability test and CO_2 selectivity of Ru_1/CeO_2 and Ru_n/CeO_2 catalysts.

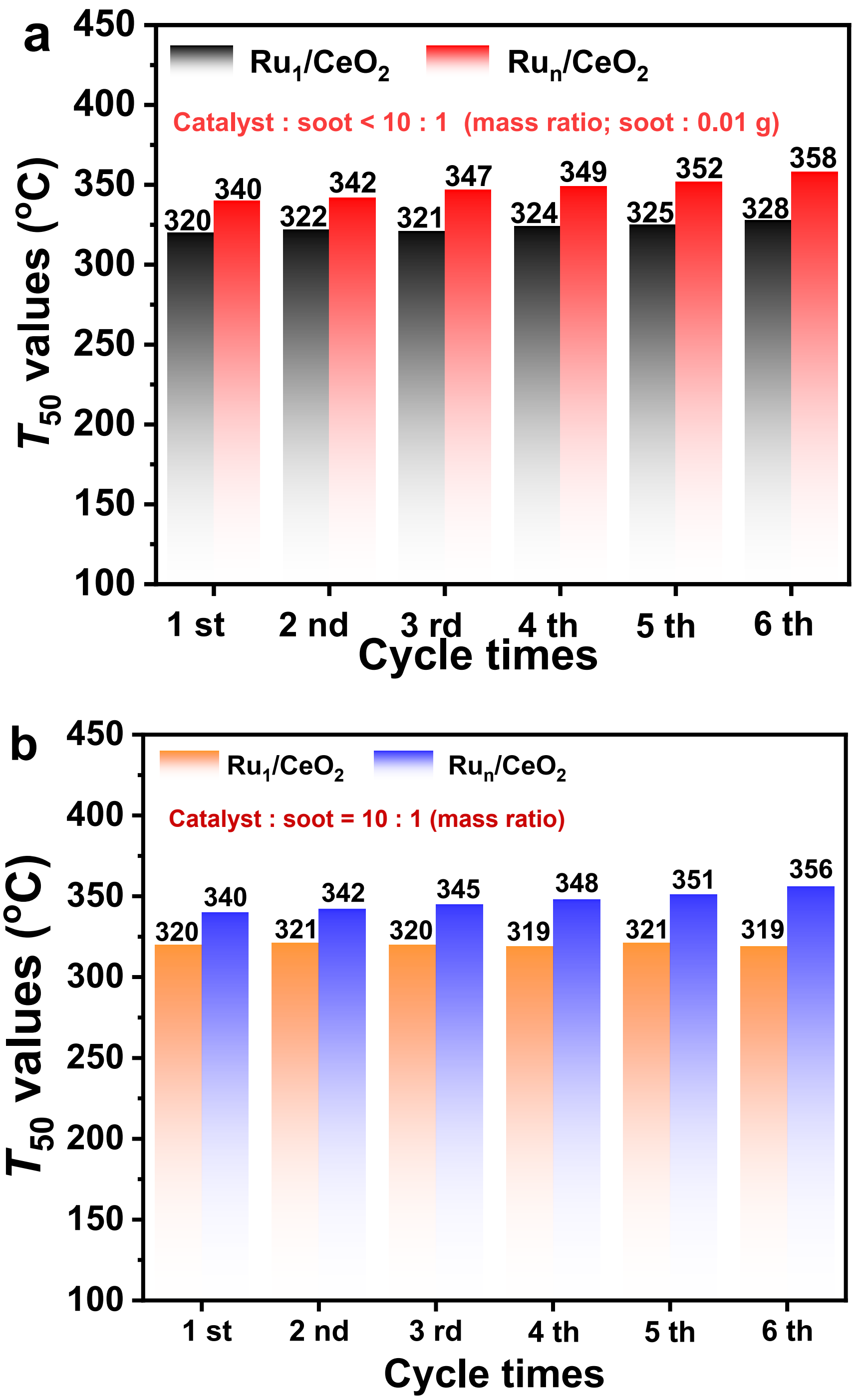

Supplementary Figure 16. Cyclic stability test of Ru_1/CeO_2 and Ru_n/CeO_2 catalysts. (a) the mass of soot remained at 0.01g and (b) the mass ratio of catalyst to soot (< 0.01 g) kept at 10:1 for each test during six cycles of soot-TPO.

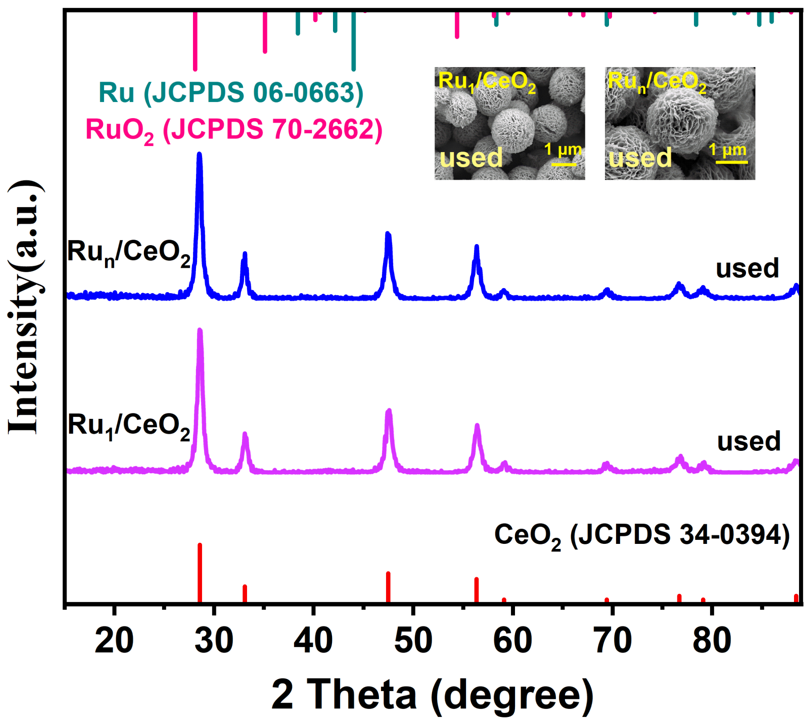

Supplementary Figure 17. XRD and SEM images of Ru_1/CeO_2 and Ru_n/CeO_2 catalysts after six times soot-TPO cycle.

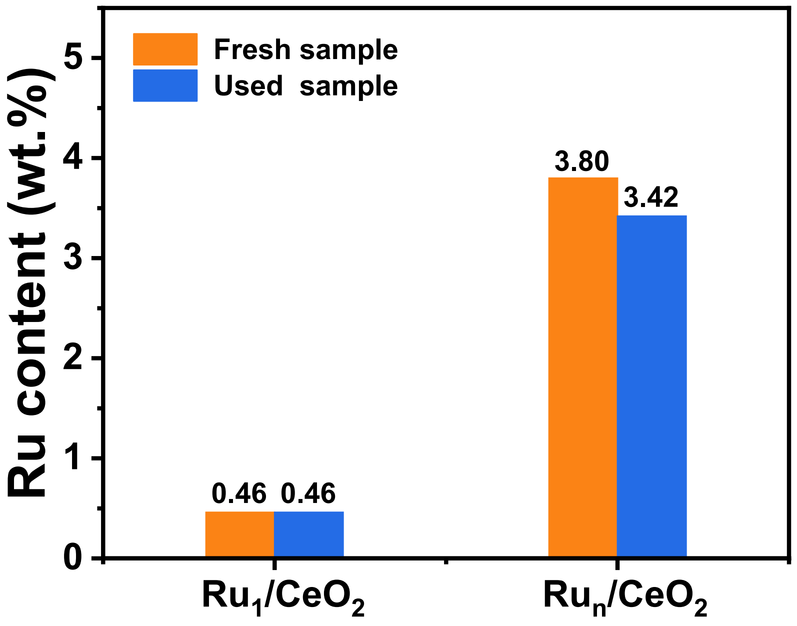

Supplementary Figure 18. ICP-OES results of Ru_1/CeO_2 and Ru_n/CeO_2 catalysts after six times soot-TPO cycle.

Note: To investigate the stability of Ru species during soot oxidation, the inductively coupled plasma optical emission spectroscopy (ICP-OES) was utilized to determine Ru content for the used Ru_1/CeO_2 and used Ru_n/CeO_2 catalysts. The ICP-OES analysis of the used Ru_1/CeO_2 catalyst (0.46 wt.%) indicated no loss of Ru even after six cycles below 450 °C. However, the Ru content of Ru_n/CeO_2 (3.42 wt.%) declined after six cycles contrasted with the fresh Ru_n/CeO_2 catalyst (3.80 wt.%). The Ru content on the Ru_n/CeO_2 catalyst has significantly reduced, which could be attributed to the volatilization of Ru species. Thus, Ru_1/CeO_2 catalyst with the surface lattice confinement single atom Ru can inhibit the volatilization of Ru species to be highly stable.

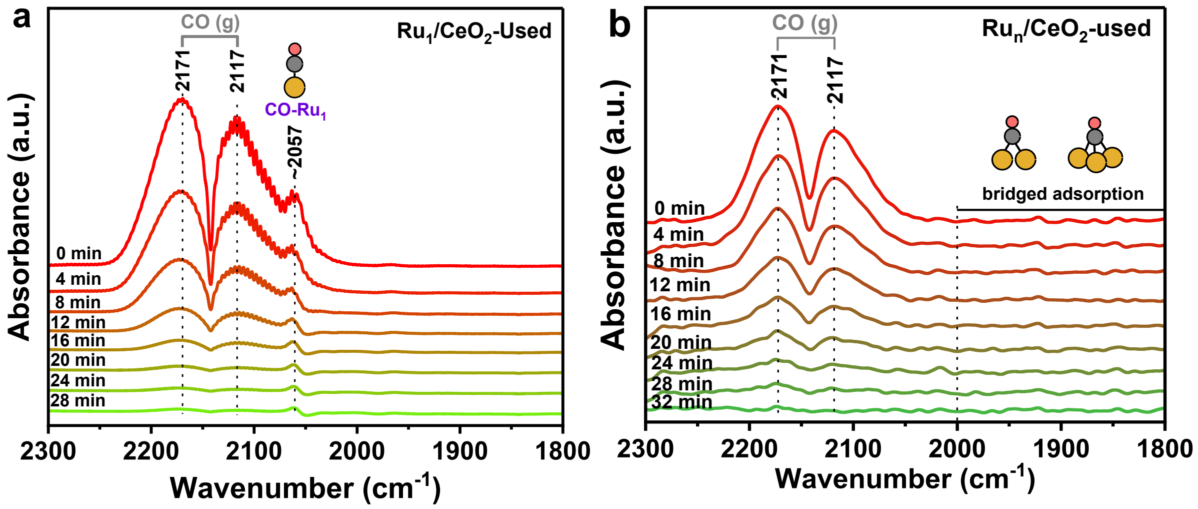

Supplementary Figure 19. DRIFT spectra of CO adsorption for the used Ru_1/CeO_2 (a) and Ru_n/CeO_2 (b) catalysts.

Note: The peaks at 2170 and 2117 cm^{-1} can be assigned to the R and P branches of the rotation vibrational spectra of gas-phase CO. The strongest peak centered at ~2057 cm^{-1} is ascribed to the C-O stretching vibration of dicarbonyl CO species ($Ru^{n+}(CO)_2$) adsorption on single atomically dispersed Ru^{n+} sites. The adsorption peak at ~1830 and ~1860 cm^{-1} can be assigned to the bridged adsorption of CO on two and three Ru atoms. For used Ru_1/CeO_2 catalyst, the adsorption peaks at 2170, 2117, and 2057 cm^{-1}, and the adsorption peaks at 1830 and 1860 cm^{-1} are undetected. It indicates that the Ru species in used Ru_1/CeO_2 catalyst maintain single atomically dispersed ionic state, which can be attributed to the lattice-confined Ru single atom catalyst formation of the Ru-O-Ce bond, enhancing the interaction between Ru species and CeO_2. Whereas the used Ru_n/CeO_2 catalyst shows weak peaks at the range of 1800-2000 cm^{-1}, suggesting the bridged adsorption of CO on two and three Ru atoms.

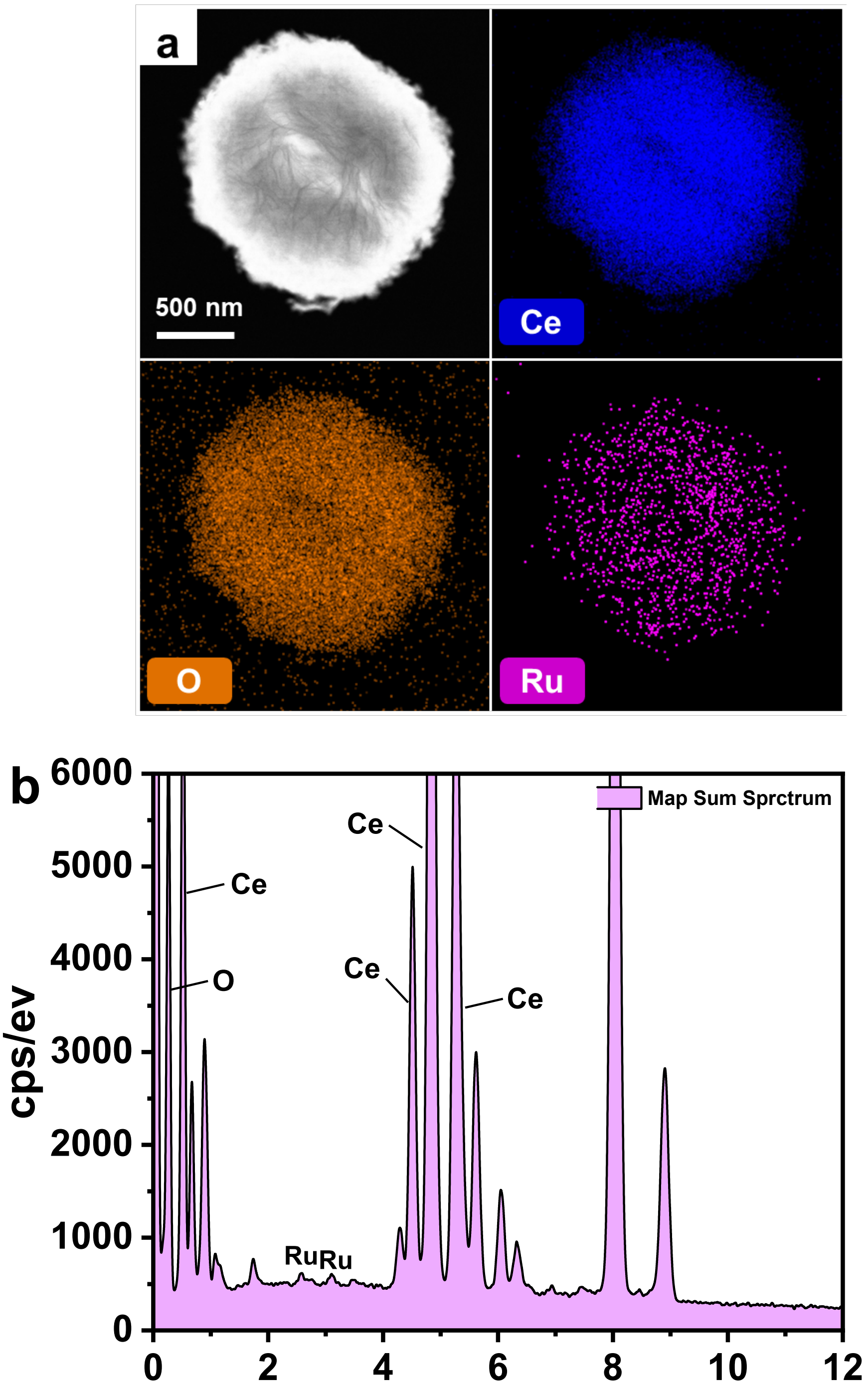

Supplementary Figure 20. (a) STEM images and corresponding EDX mapping and (b) map sum spectrum date image over Ru_1/CeO_2 catalyst after six-time soot-TPO tests.

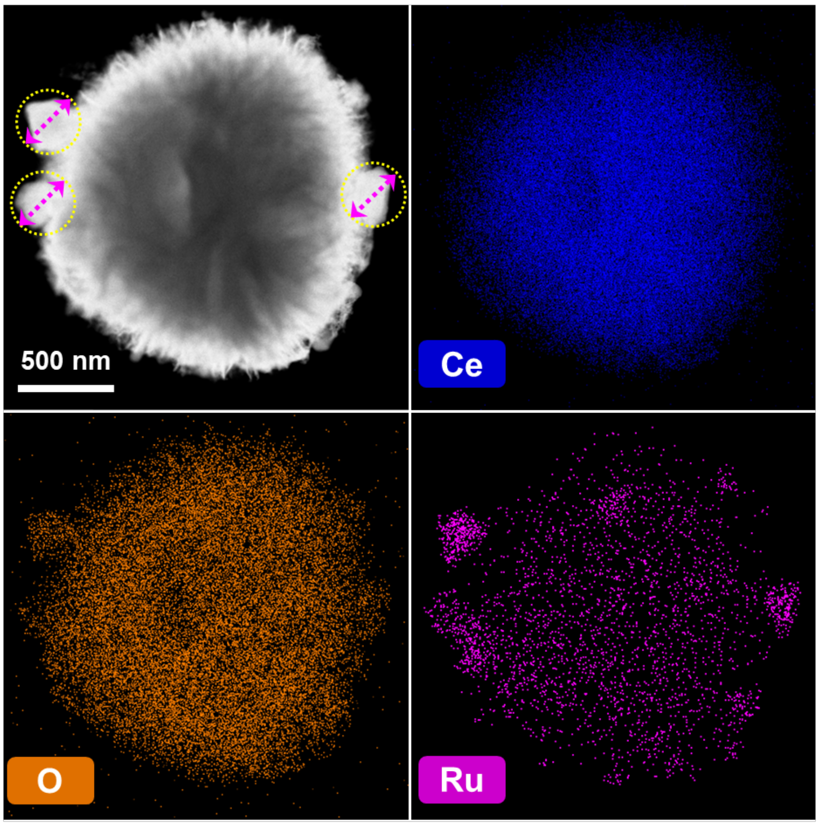

Supplementary Figure 21. STEM images over Ru_n/CeO_2 catalyst and corresponding EDX mapping after six-time soot-TPO tests.

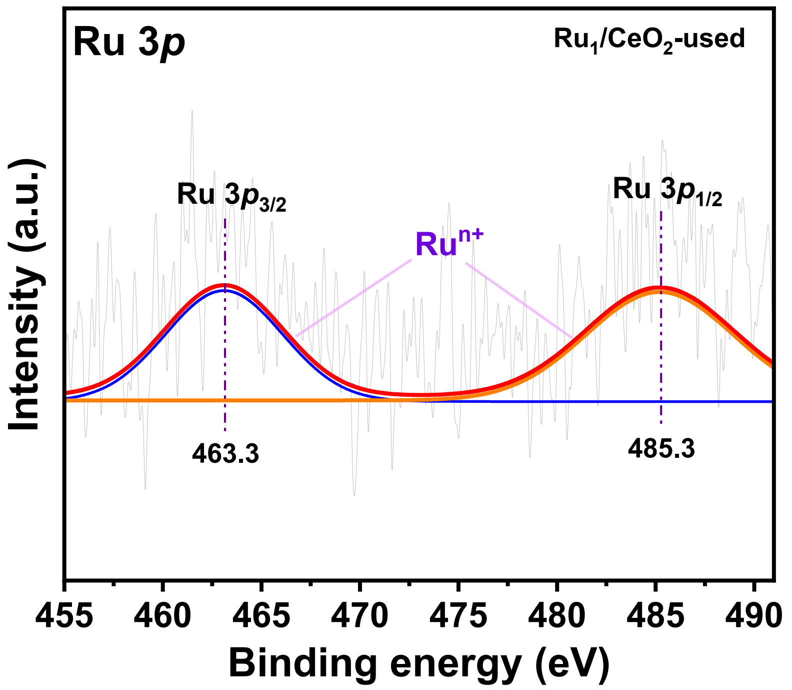

Supplementary Figure 22. XPS spectra of Ru 3*p* for used Ru_1/CeO_2 catalyst.

.

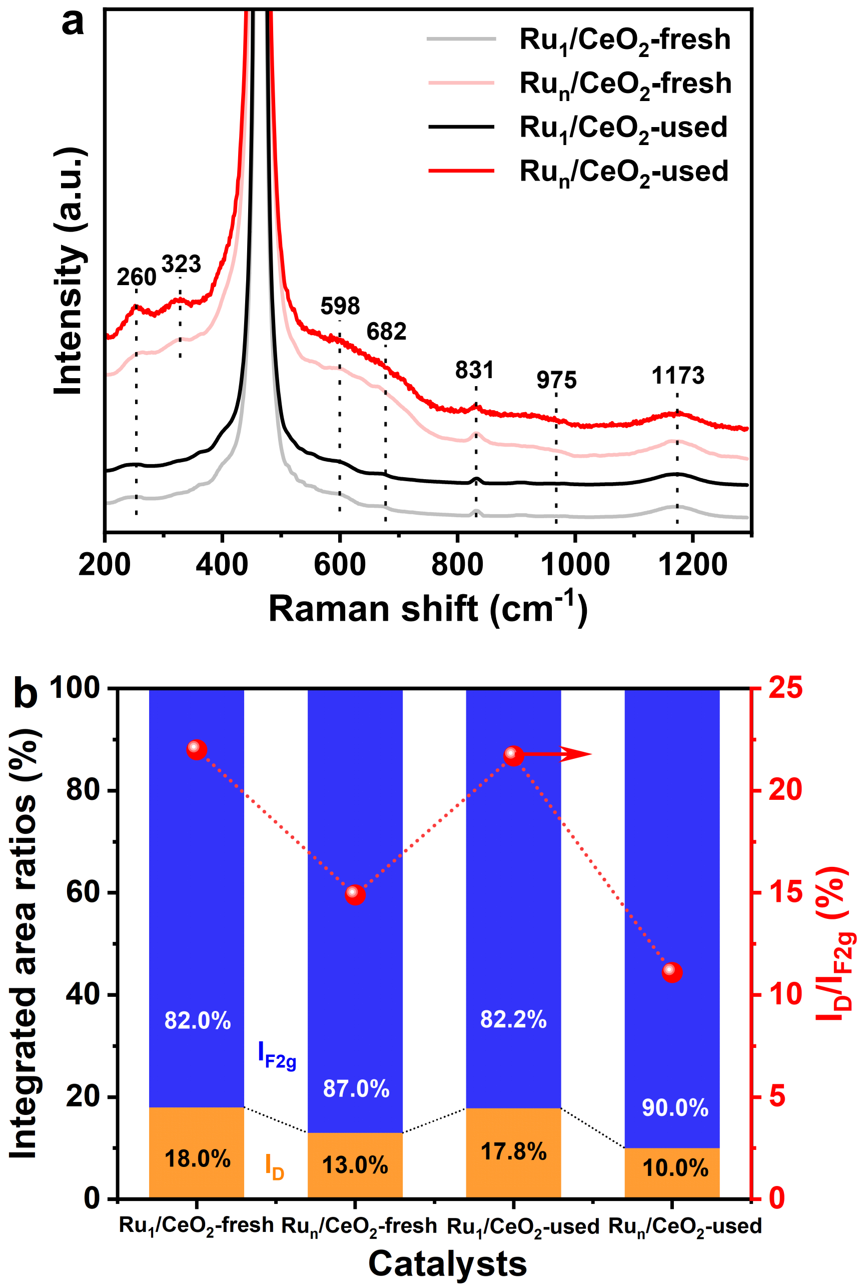

Supplementary Figure 23. (a) Raman spectra and (b) the integrated area ratios of *D* to F_{2g} peaks (I_D/I_{F2g}) in Raman spectra for of Ru_1/CeO_2 and Ru_n/CeO_2 catalysts after six cycles of soot-TPO tests.

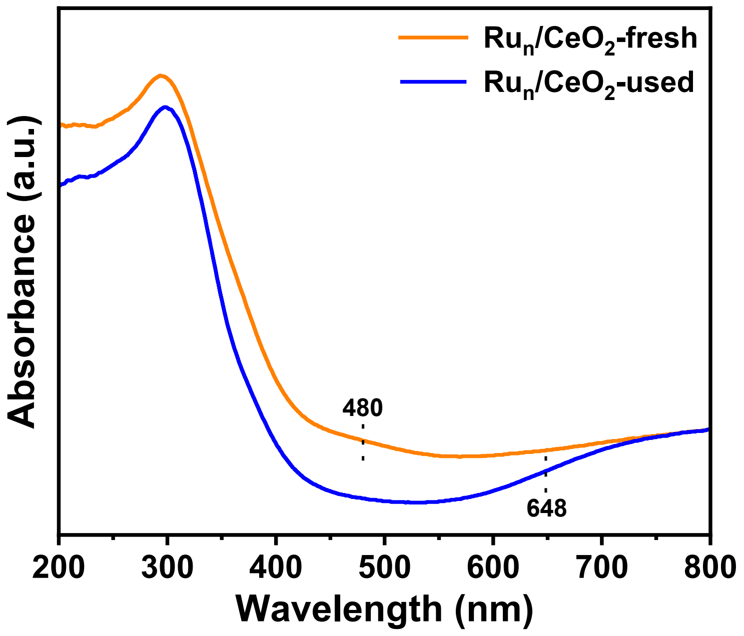

Supplementary Figure 24. UV-Vis spectra of fresh and used Ru_n/CeO_2 catalysts.

Note: The peak at 400-700 nm can be reasonably assigned to the surface plasmon resonance (SPR) of RuO_2 nanoparticles supported on CeO_2. The SPR peak red-shift of RuO_2 nanoparticles increases with their particle size. Therefore, the average size of RuO_2 nanoparticles increased during the soot oxidation.

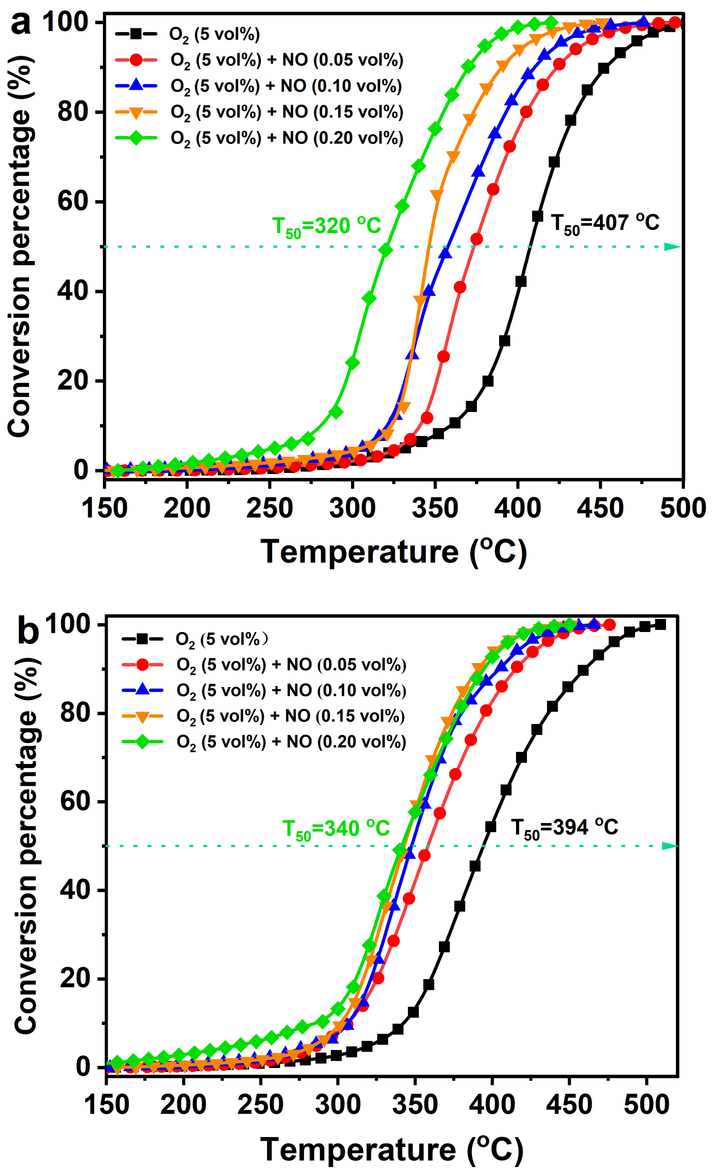

Supplementary Figure 25. Conversion percentage of soot oxidation over Ru_1/CeO_2 (a) and Ru_n/CeO_2 (b) catalysts under different NO concentration.

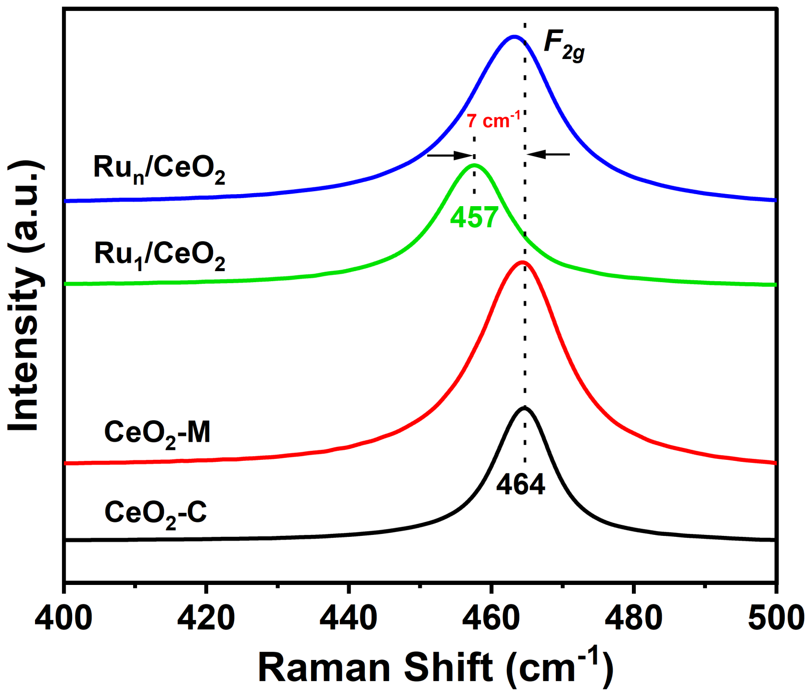

Supplementary Figure 26. The partially magnified Raman spectra of all as-prepared catalysts.

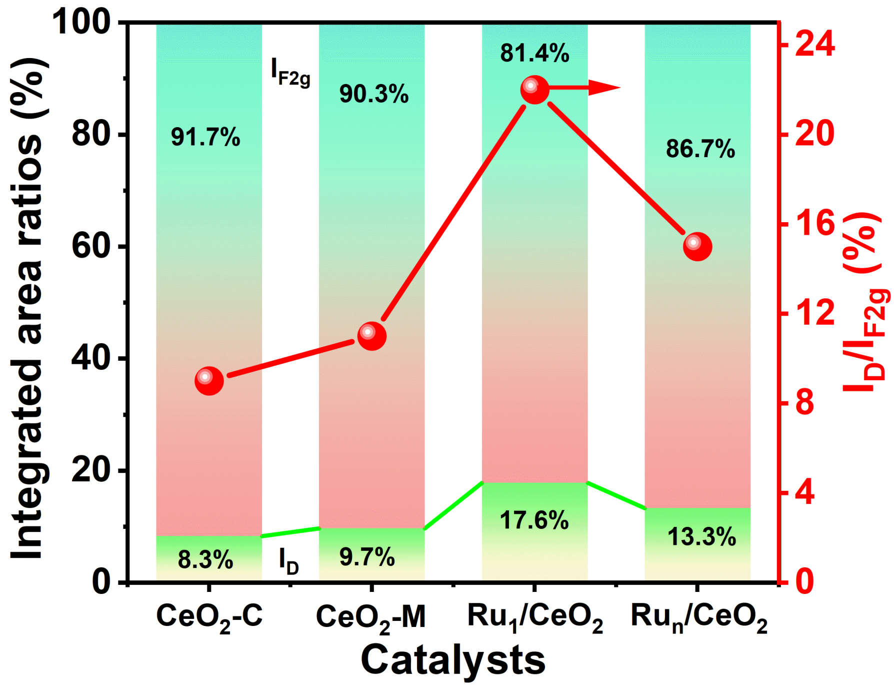

Supplementary Figure 27. The integrated area ratios of *D* to F_{2g} peaks (I_D/I_{F2g}) in Raman spectra for all as-prepared catalysts.

Note: For CeO_2 catalyst, there are a single strong peak at 464 cm^{-1} and three weak peaks at 260, 598, and 1173 cm^{-1} that can be assigned to the F_{2g} mode, second-order transverse acoustic (*2TA*) mode, defect-induced (*D*) mode, and second-order longitudinal optical (*2LO*) mode of the fluorite phase, respectively. The ratio between the integrated area of the *D* peak and the F_{2g} peak (marked as I_D/I_{F2g}) represents the relative concentration of oxygen vacancy on the CeO_2 surface.

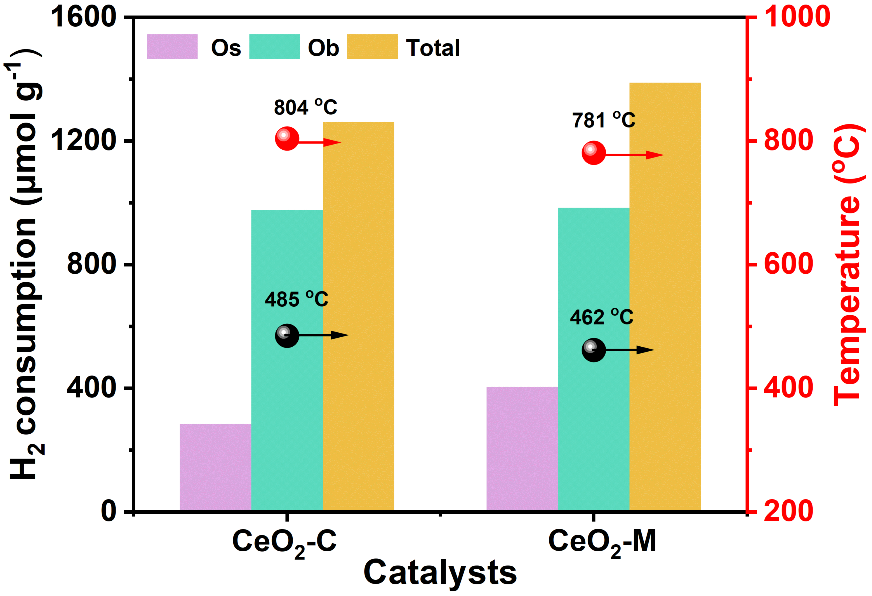

Supplementary Figure 28. The H_2 consumption corresponds to oxygen species for CeO_2-C and CeO_2-M catalysts: the Os represents the surface oxygen, and the Ob represents the bulk oxygen.

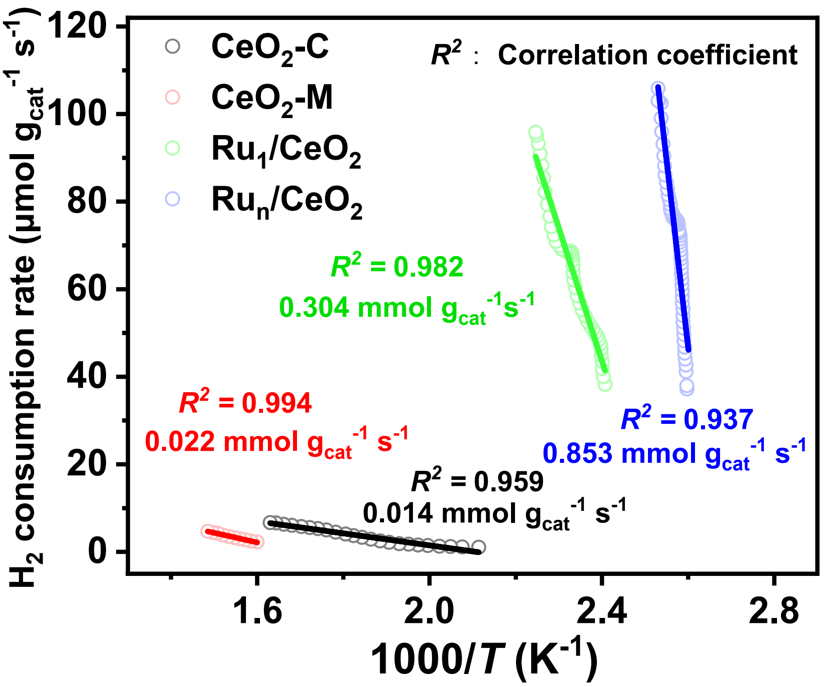

Supplementary Figure 29. The initial H_2 consumption rate as a function of inverse temperature over all as-prepared catalysts.

Note: The reducibility of a catalyst can be effectively evaluated by the initial H_2 consumption rate (where less than 25% oxygen in the catalyst was consumed for the first reduction peak). H_2 consumption was quantified by a CuO sample as a reference. The initial H_2 consumption can be obtained by the following method: Firstly, the time, temperature and correspondingly cumulative H_2 consumption (less than 25% of first reduction peak) were calculated and selected. Next, the H_2 consumption rate can be calculated by the ratio of cumulative H_2 consumption to time. Finally, the curve of the initial H_2 consumption rate can be obtained which takes 1000/(T+273.15) as the abscissa and the H_2 consumption rate as the ordinate.

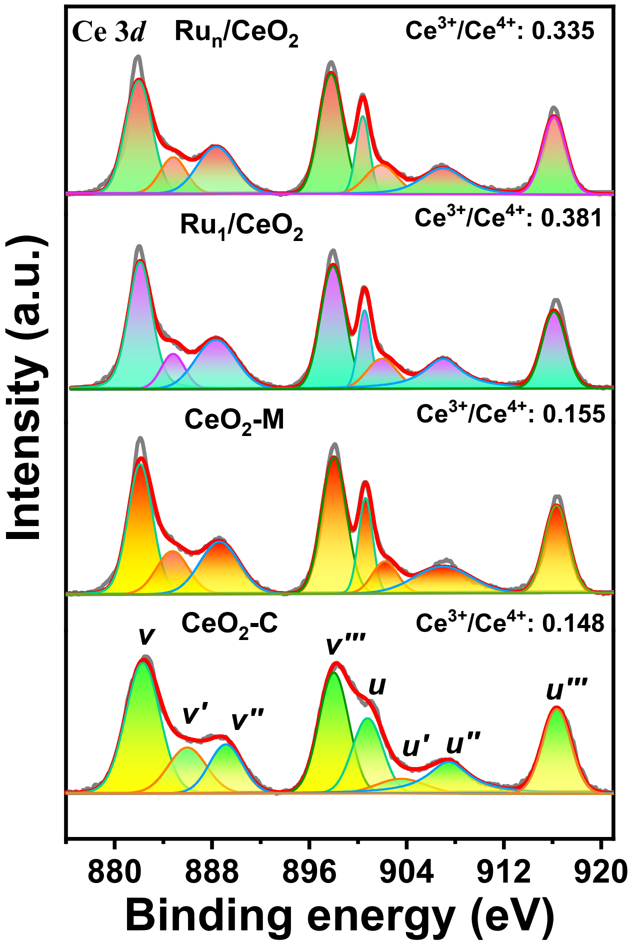

Supplementary Figure 30. Ce 3*d* XPS curves of the all as-prepared catalysts.

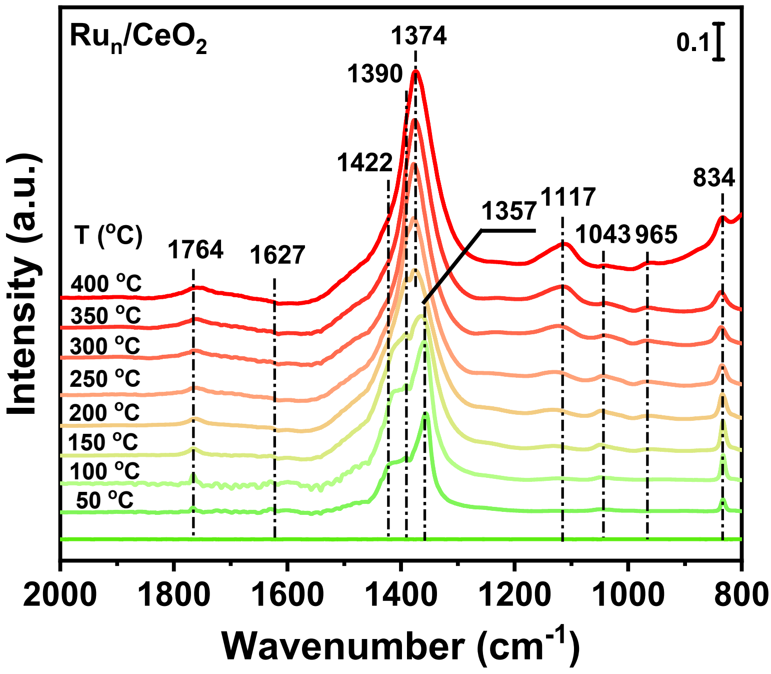

Supplementary Figure 31. Temperature-dependent *in-situ* DRIFTS spectra of NO oxidation on Ru_n/CeO_2 catalyst.

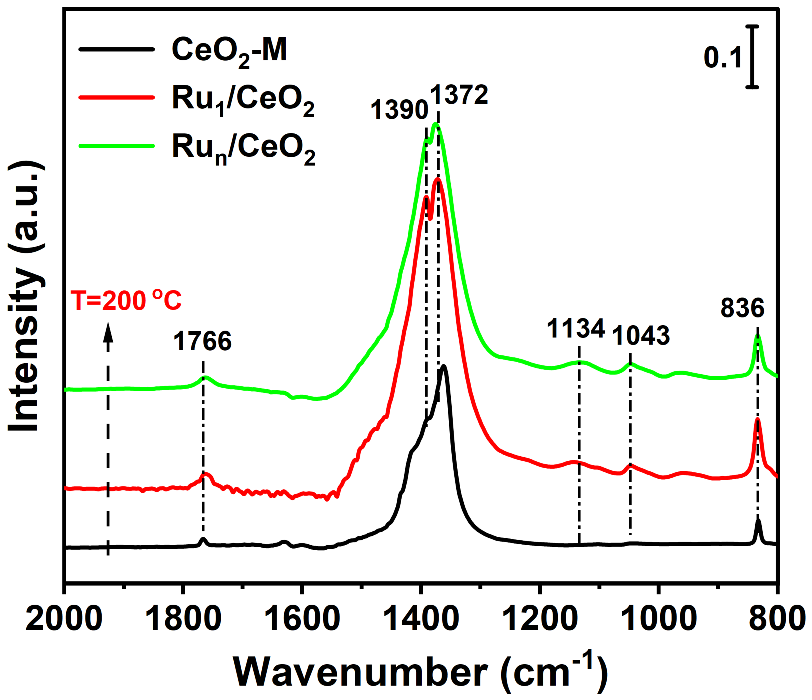

Supplementary Figure 32. *In-situ* DRIFTS results of CeO_2-M, Ru_1/CeO_2, and Ru_n/CeO_2 from 2000 to 800 cm^{-1}, the sample were detected at 200 °C under 50 mL min^{-1} (5 vol% O_2, 0.2 vol% NO balanced with N_2).

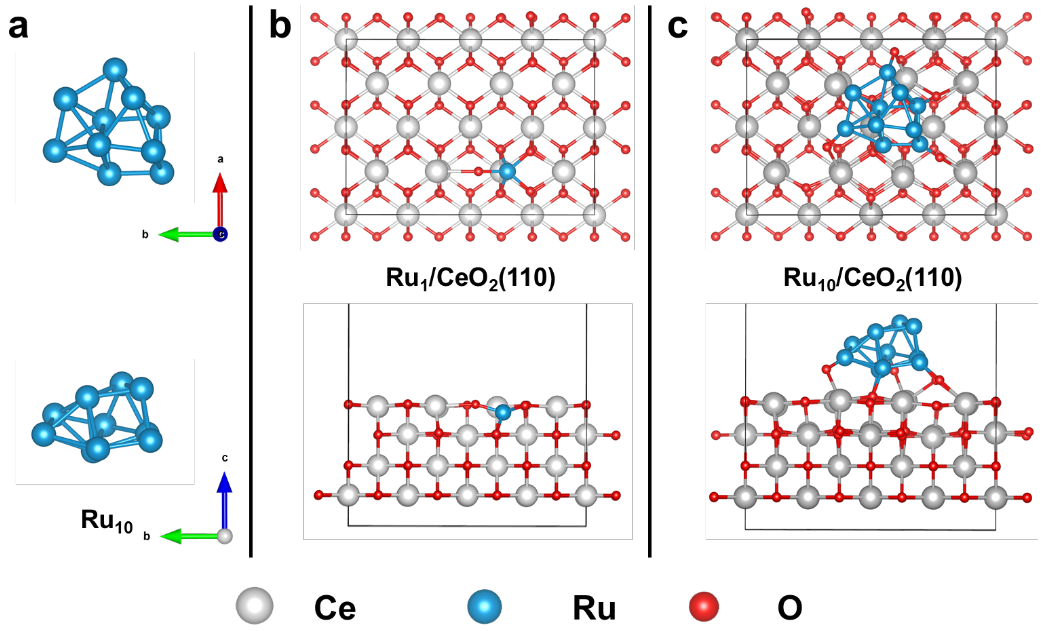

Supplementary Figure 33. The optimized configurations in top and side views (a) Ru_{10}, (b) $Ru_1/CeO_2(110)$, (c) $Ru_{10}/CeO_2(110)$.

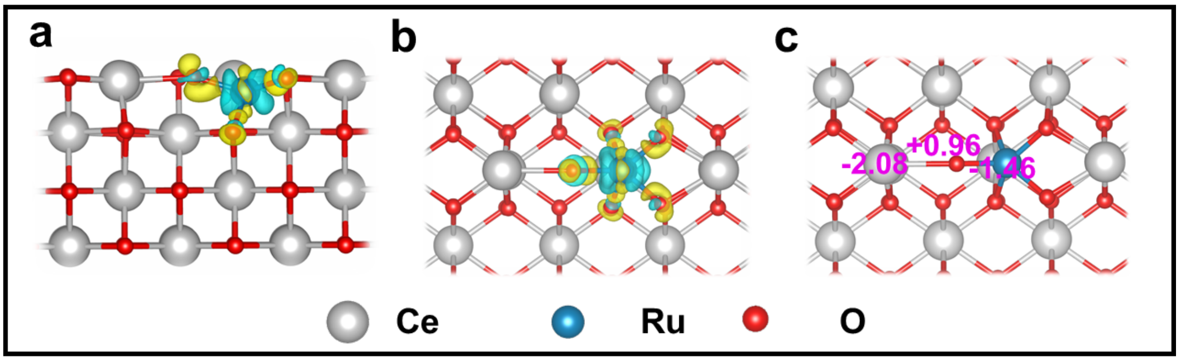

Supplementary Figure 34. Charge density difference of Ru_1/CeO_2 catalyst. (a) Side view, (b) Top view. Bader charges (c) over Ru_1/CeO_2 catalyst (Cyan and yellow regions represent electron depletion and electron accumulation, respectively. The value of the iso-surface is 0.015 e $Å^{-3}$).

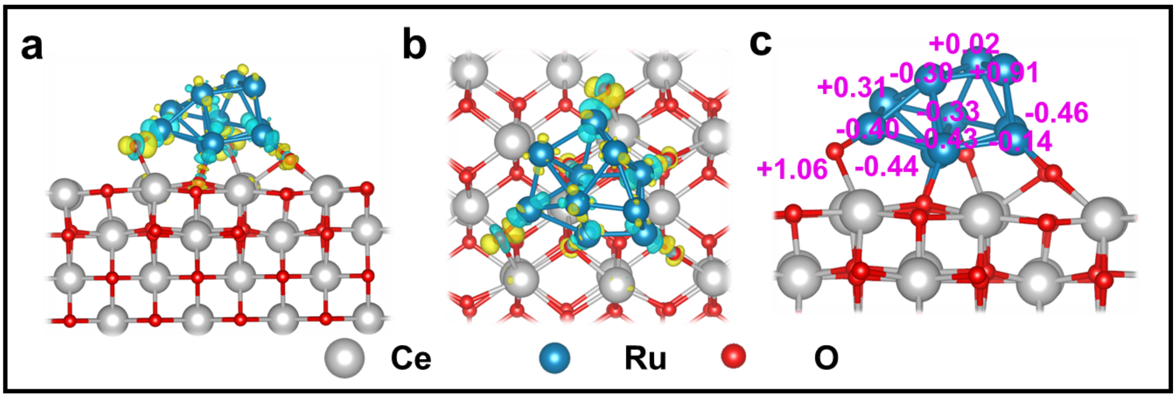

Supplementary Figure 35. Charge density difference of Ru_{10}/CeO_2 catalyst. (a) Side view, (b) Top view. Bader charges (c) over Ru_n/CeO_2 catalyst (Cyan and yellow regions represent electron depletion and electron accumulation, respectively. The value of the iso-surface is 0.015 e $Å^{-3}$).

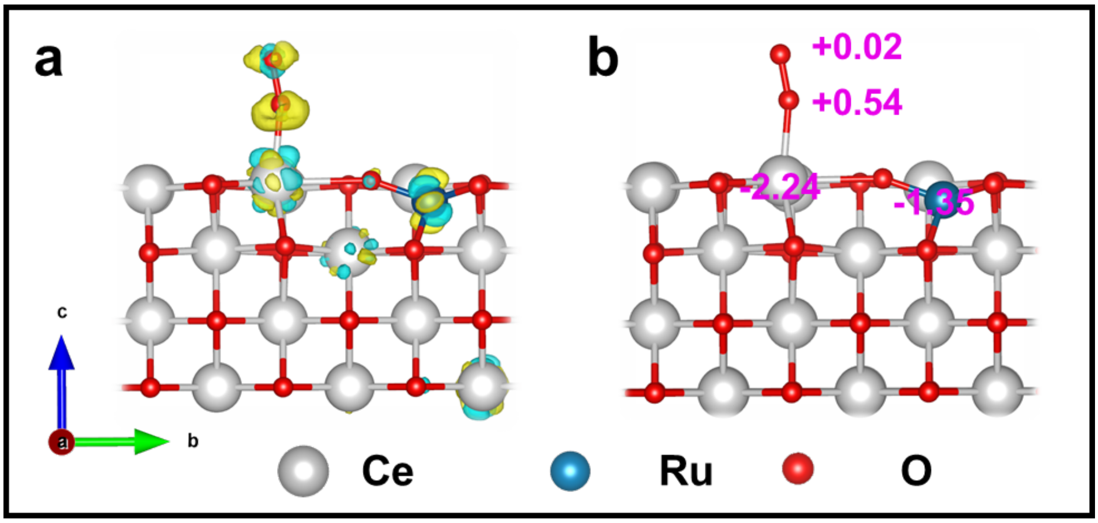

Supplementary Figure 36. Differential charge density map (a) increase (yellow color) and decrease (cyan color) of electron distribution and bader charge analyses (b) for Ru_1/CeO_2 catalyst. The value of the iso-surface is 0.01 e Å^{-3}.

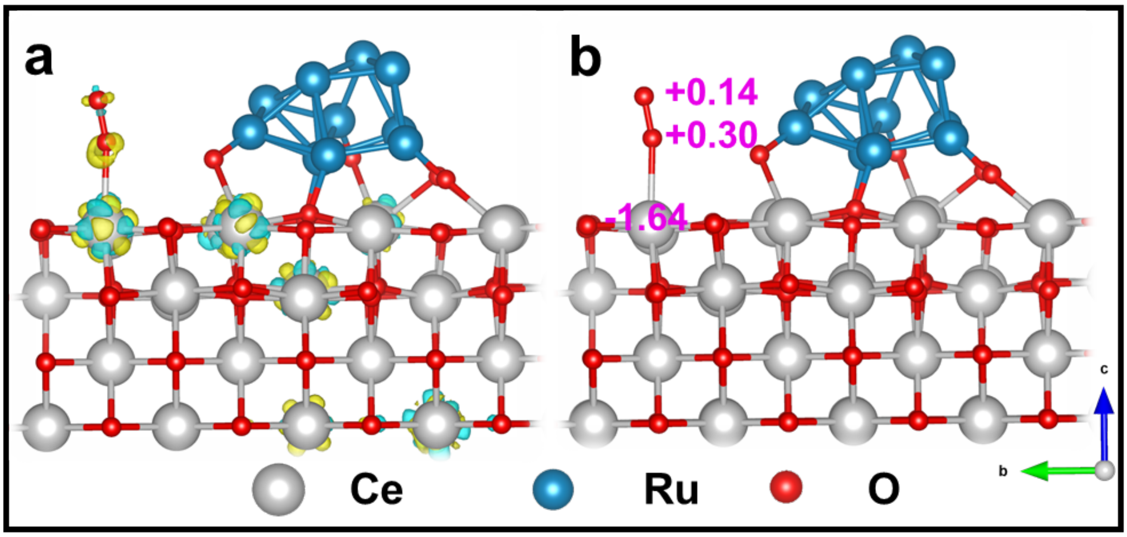

Supplementary Figure 37. Differential charge density map (a) increase (yellow color) and decrease (cyan color) of electron distribution and bader charge analyses (b) for Ru_{10}/CeO_2 catalyst. The value of the iso-surface is 0.01 e Å^{-3}.

Supplementary Tables

Supplementary Table 1. Physicochemical properties of all as-prepared catalysts. Specific surface area (S_{BET}), pore volume (V_p), pore diameter (D_p), Ru content, crystal size and lattice parameter (a) of CeO_2-C, CeO_2-M, Ru_1/CeO_2 and Ru_n/CeO_2 catalysts.

Catalysts	S_{BET} [a] (m^2/g)	V_p [b] (cm^3/g)	D_p [c] (nm)	Ru content [d] (wt %)	Crystal size [e] (nm)	a [f] (Å)
CeO_2-C	40.9	0.149	7.8	-	26.9	5.4103
CeO_2-M	58.3	0.277	10.7	-	13.1	5.4101
Ru_1/CeO_2	55.3	0.265	10.5	0.46	13.1	5.4084
Ru_n/CeO_2	49.4	0.263	10.4	3.80	13.4	5.4096

[a] Surface area was calculated by the BET method.

[b] Pore volume obtained by the BET method.

[c] Pore diameter was calculated via the BJH method according to the N_2 desorption isotherms.

[d] The Ru content obtained by ICP-OES.

[e, f] The crystal size and lattice parameter by XRD.

Supplementary Table 2. Results of EXAFS curves-fitting. Structure parameter of the Ru_1/CeO_2, Ru_n/CeO_2, Ru foil and RuO_2 at EXAFS curves-fitting at Ru K-edge.

**Sample**	**Path**	**CN**	***R* (Å)**	**σ^2 (10^{-3}Å^2)**	**ΔE_0 (eV)**	***R* factor**
Ru foil	Ru-Ru	12.0	2.68 ± 0.003	3.3 ± 0.4	4.5 ± 0.8	0.008
RuO_2	Ru-O	6.0	1.95 ± 0.011	3.2 ± 1.1	8.4 ± 1.4	0.014
	Ru-Ru	4.0	3.14 ± 0.016	3.2 ± 1.1	8.4 ± 1.4	0.014
Ru_1/CeO_2	Ru-O	5.3 ± 0.4	2.01 ± 0.005	5.2 ± 0.3	0.5 ± 0.3	0.006
Ru_n/CeO_2	Ru-O	3.4 ± 0.9	2.04 ± 0.003	6.1 ± 0.2	-4.8 ± 1.2	0.009
	Ru-Ru	4.2 ± 0.3	2.62 ± 0.004	5.3 ± 0.3	-4.8 ± 1.2	0.009

CN is the coordination number; *R* is the bond length; σ^2 is the Debye-Waller factor; ΔE_0 is the inner potential correction; *R*-factor (%) indicates the goodness of the fit. S_0^2 was fixed to 0.75 for catalysts.

Supplementary Table 3. Comparison of catalytic properties. Comparison of soot oxidation catalyst system, reaction condition, catalytic active (T_{50}) and corresponding reference.

Catalyst	Reaction Condition	T_{50} (°C)	Ref.
**Ru_1/CeO_2**	**2000 ppm NO/5% O_2 /Ar (50 mL min^{-1})**	**320**	**This work**
**Ru_n/CeO_2**	**2000 ppm NO/5% O_2 /Ar (50 mL min^{-1})**	**340**	
1% Pt/Al_2O_3 (Commercial)	500 ppm NO_x/5% O_2/N_2 (500 mL min^{-1})	475	1
Pt/Al_2O_3	1000 ppm NO/10% O_2/N_2 (500 mL min^{-1})	464	2
3DOMM $PdCo_2O_4$/CZO	2000 ppm NO/5% O_2/Ar (50 mL min^{-1})	367	3
Ag/Co-LDO-H	600 ppm NO/10% O_2/N_2 (150 mL min^{-1})	346	4
$Au_6@La_2O_3$/LOC-R	2000 ppm NO/ 5% O_2/ Ar (50 mL min^{-1})	372	5
AgCe-NC	1% O_2/N_2 (500 mL min^{-1})	376	6
$Pt/Ce_{50}Pr_{50}$-NP	550 ppm NO/10% O_2/N_2 (100 mL min^{-1})	456	7
$Pt@CoO_x/Al_2O_3$	2000 ppm NO/5% O_2/5% H_2O/Ar (50 mL min^{-1})	357	8
$PdAu@CeO_2$/CZ	2000 ppm NO/5% O_2/5% H_2O/Ar (50 mL min^{-1})	363	9
Au/CeO_2-R	2000 ppm NO/ 5% O2/ Ar (50 mL min-1)	350	10

$LCP_{0.06}$-4	Air	403	11
Pt-$KMnO_x/Ce_{0.25}Zr_{0.75}O_2$	2000 ppm NO/ 5% O_2/ Ar (50 mL min^{-1})	330	12
$LA_{2.5}C$	2000 ppm NO/10% O_2/N_2 (50 mL min^{-1})	358	13
Pt/Fe_2O_3-19	2000 ppm NO/5% O_2/5% H_2O/Ar (50 mL min^{-1})	365	14
Ag/TiO_2-79%	2000 ppm NO/ 5% O_2/ Ar (50 mL min^{-1})	364	15
Pt/CeO_2-NC	550 ppm NO+10% O_2/N_2 (50 mL min^{-1})	484	16
Pt/MnO_x-CeO_2	2000 ppm NO/5% O_2/5% H_2O/N_2 (500 mL min^{-1})	520	17
$Pt_{2.0}$@$CeO_{2-\delta}/CeO_2$	2000 ppm NO/ 5% O_2/ Ar (50 mL min^{-1})	330	18

Supplementary Table 4. Apparent activation energy. The Arrhenius plots of $\ln\left[-\frac{\ln(1-\alpha)}{T^2}\right]$ versus $1/T$ for all catalysts and obtained E_a values and the values of the Correlation Coefficients (R^2), and the values of Pre-Exponential Factor (A) of CeO_2-C, CeO_2-M, Ru_1/CeO_2, and Ru_n/CeO_2 catalysts.

**Catalysts**	**Slop**	**Correlation Coefficients (R^2)**	**Pre-Exponential Factors (A)**	**Apparent Activation Energy (E_a)**
			**s^{-1}**	**$kJ\ mol^{-1}$**
CeO_2-C	15.4	0.997	4.32×10^6	128.0
CeO_2-M	13.3	0.998	2.24×10^5	110.6
Ru_1/CeO_2	9.04	0.999	6.22×10^4	75.2
Ru_n/CeO_2	11.1	0.999	1.24×10^4	92.3

Supplementary Table 5. Results of XPS. Surface compositions and oxidation states of Ru (3*p*), Ce (3*d*) and O (1*s*) species for all catalysts derived from XPS analysis.

Catalysts	Ru species (%)			Ce species (%)			O species (%)			
	Ru^0	Ru^{n+}	R^a	Ce^{3+}	Ce^{4+}	R^b	O^{2-}	O_2^{2-}	O_2^-	R^c
CeO_2-C	-	-	-	12.9	87.1	0.148	72.5	17.2	10.3	0.379
CeO_2-M	-	-	-	13.4	86.6	0.155	68.9	20.4	10.7	0.451
Ru_1/CeO_2	0	100	0	27.6	72.4	0.381	62.5	28.1	9.4	0.600
Ru_n/CeO_2	54.0	46.0	1.17	25.1	74.9	0.335	66.3	25.6	8.1	0.508

[a] The Ru species ratio Ru^0/Ru^{n+}.

[b] The Ce species ratio Ce^{3+}/Ce^{4+}.

[c] The ratio of the absorbed oxygen ($O_2^- + O_2^{2-}$) to lattice oxygen (O^{2-}).

Supplementary Table 6. Location and types of peaks. Assignments of the bands during temperature-dependent in situ DRIFTS spectra of NO oxidation over Ru/CeO_2 catalyst.

**Wavenumbers (cm^{-1})**	**Species**	**Assignment**	**References**
~1766	N_2O_4 dimer	v_{as} (N-O)	19
~1627	bridging nitrates	v_{as} (NO_3^-) split	20
~1422	monodentate nitrites	v (N=O)	21
~1390	ionic nitrites	v_{as}($M^+[NO_2]$)	22
~1372	ionic nitrates	v_{as} (NO_3^-)	23
~1360	monodentate nitrite	v (M-NO_2)	24
~1134	anionic nitrates	v_1	25
~1117	nitrites	v_1	26
~1043	bidentate nitrates	v_1	25,26
~965	chelating bidentate NO_3^-	v_3	27
~836	bending vibration of nitrates	v_2	28,29

v_1: symmetric stretching vibration; v_2: out-of-plane bending vibration; v_3: double degenerate vibration; v_{as}: asymmetric stretching vibration.

Supplementary References

1. Guillén-Hurtado N, García-García A, Bueno-López A. Active oxygen by Ce–Pr mixed oxide nanoparticles outperform diesel soot combustion Pt catalysts. *Appl. Catal. B* **174-175**, 60-66 (2015).
2. Liu S, Wu X, Weng D, Li M, Fan J. Sulfation of Pt/Al_2O_3 catalyst for soot oxidation: High utilization of NO_2 and oxidation of surface oxygenated complexes. *Appl. Catal. B* **138-139**, 199-211 (2013).
3. Xiong J, et al. Fabrication of spinel-type $Pd_xCo_{3-x}O_4$ binary active sites on 3D ordered meso-macroporous Ce-Zr-O_2 with enhanced activity for catalytic soot oxidation. *ACS Catal.* **8**, 7915-7930 (2018).
4. Ren W, et al. Identifying oxygen activation/oxidation sites for efficient soot combustion over silver catalysts interacted with nanoflower-like hydrotalcite-derived CoAlO metal oxides. *ACS Catal.* **9**, 8772-8784 (2019).
5. Wu Q, et al. Interaction-induced self-assembly of Au@La_2O_3 core–shell nanoparticles on $La_2O_2CO_3$ nanorods with enhanced catalytic activity and stability for soot oxidation. *ACS Catal.* **9**, 3700-3715 (2019).
6. Liu S, et al. Soot oxidation over CeO_2 and Ag/CeO_2: Factors determining the catalyst activity and stability during reaction. *J Catal*. **337**, 188-198 (2016).
7. Andana T, et al. Nanostructured equimolar ceria-praseodymia for NO_x-assisted soot oxidation: Insight into Pr dominance over Pt nanoparticles and metal–support interaction. *Appl. Catal. B* **226**, 147-161 (2018).
8. Wu Q, et al. High-efficient catalysts of core-shell structured Pt@transition metal oxides (TMOs) supported on 3DOM-Al_2O_3 for soot oxidation: The effect of strong Pt-TMO interaction. *Appl. Catal. B* **244**, 628-640 (2019).
9. Xiong J, et al. Efficiently multifunctional catalysts of 3D ordered meso-macroporous

$Ce_{0.3}Zr_{0.7}O_2$-supported PdAu@CeO_2 core-shell nanoparticles for soot oxidation: Synergetic effect of Pd-Au-CeO_2 ternary components. *Appl. Catal. B* **251**, 247-260 (2019).

10. Wei Y, et al. Boosting the removal of diesel soot particles by the optimal exposed crystal facet of CeO_2 in Au/CeO_2 catalysts. *Environ. Sci. Technol.* **54**, 2002-2011 (2020).
11. Zeng L, Cui L, Wang C, Guo W, Gong C. In-situ modified the surface of Pt-doped perovskite catalyst for soot oxidation. *J. Hazard. Mater.* **383**, 121210 (2020).
12. Xiong J, et al. Synergetic effect of K sites and Pt nanoclusters in an ordered hierarchical porous Pt-$KMnO_x$/$Ce_{0.25}Zr_{0.75}O_2$ catalyst for boosting soot oxidation. *ACS Catal.* **10**, 7123-7135 (2020).
13. He L, et al. Promotion of A-site Ag-doped perovskites for the catalytic oxidation of soot: Synergistic catalytic effect of dual active sites. *ACS Catal.* **11**, 14224-14236 (2021).
14. Li Y, et al. Facilitating catalytic purification of auto-exhaust carbon particles via the Fe_2O_3{113} facet-dependent effect in Pt/Fe_2O_3 catalysts. *Environ. Sci. Technol.* **55**, 16153-16162 (2021).
15. Zhang P, et al. Exposed {001} facet of anatase TiO_2 nanocrystals in Ag/TiO_2 catalysts for boosting catalytic soot combustion: The facet-dependent activity. *J Catal* **398**, 109-122 (2021).
16. Andana T, et al. Ceria-supported small Pt and Pt_3Sn nanoparticles for NO_x-assisted soot oxidation. *Appl. Catal. B* **209**, 295-310 (2017).
17. Zhang H, Yuan S, Wang J, Gong M, Chen Y. Effects of contact model and NO_x on soot oxidation activity over Pt/MnO_x-CeO_2 and the reaction mechanisms. *Chem. Eng. J.* **327**, 1066-1076 (2017).
18. Wei Y, Zhao Z, Liu J, Xu C, Jiang G, Duan A. Design and synthesis of 3D ordered macroporous CeO_2-supported Pt@$CeO_{2-\delta}$ core–shell nanoparticle materials for enhanced catalytic activity of soot oxidation. *Small* **9**, 3957-3963 (2013).

19. Niu H, Li K, Chu B, Su W, Li J. Heterogeneous reactions between toluene and NO_2 on mineral particles under simulated atmospheric conditions. *Environ. Sci. Technol.* **51**, 9596-9604 (2017).

20. Shang H. et al. Oxygen vacancies promoted the selective photocatalytic removal of NO with blue TiO_2 via simultaneous molecular oxygen activation and photogenerated hole annihilation. *Environ. Sci. Technol.* **53**, 6444-6453 (2019).

21. Wang H, Li K, Li J, Sun Y, Dong F. Photochemical transformation pathways of nitrates from photocatalytic NO_x oxidation: Implications for controlling secondary pollutants. *Environ. Sci. Technol. Lett.* **8**, 873-877 (2021).

22. Urán L, Gallego J, Ruiz W, Bailón-García E, Bueno-López A, Santamaría A. Monitoring intermediate species formation by DRIFT during the simultaneous removal of soot and NO_x over $LaAgMnO_3$ catalyst. *Appl. Catal. A* **588**, 117280 (2019).

23. Liu Y, Meng M, Zou Z-q, Li X-g, Zha Y-q. In situ DRIFTS investigation on the NO_x storage mechanisms over Pt/K/TiO_2–ZrO_2 catalyst. *Catal. Commun.* **10**, 173-177 (2008).

24. Wang F, Shen B, Zhu S, Wang Z. Promotion of Fe and Co doped Mn-Ce/TiO_2 catalysts for low temperature NH_3-SCR with SO_2 tolerance. *Fuel* **249**, 54-60 (2019).

25. Castoldi L, Lietti L, Forzatti P, Morandi S, Ghiotti G, Vindigni F. The NO_x storage-reduction on PtK/Al_2O_3 Lean NO_x trap catalyst. *J Catal.* **276**, 335-350 (2010).

26. Li J. et al. Distribution and valence state of Ru species on CeO_2 supports: Support shape effect and its influence on CO oxidation. *ACS Catal.* **9**, 11088-11103 (2019).

27. Hadjiivanov K, Avreyska V, Klissurski D, Marinova T. Surface species formed after NO adsorption and NO + O_2 coadsorption on ZrO_2 and sulfated ZrO_2: An FTIR spectroscopic study. *Langmuir* **18**, 1619-1625 (2002).

28. Vogt R, Finlayson-Pitts BJ. A Diffuse Reflectance Infrared fourier transform spectroscopic study of the surface reaction of NaCl with gaseous NO_2 and HNO_3. *J. Phys. Chem.* **98**,

3747-3755 (1994).

29. Zhang Q-N, Zhang Y, Cai C, Guo Y-C, Reid JP, Zhang Y-H. In situ observation on the dynamic process of evaporation and cystallization of sodium nitrate droplets on a ZnSe substrate by FTIR-ATR. *J. Phys. Chem. A* **118**, 2728-2737 (2014).
